# PhyClone: accurate Bayesian reconstruction of cancer phylogenies from bulk sequencing

**DOI:** 10.1093/bioinformatics/btaf344

**Published:** 2025-06-13

**Authors:** Emilia Hurtado, Alexandre Bouchard-Côté, Andrew Roth

**Affiliations:** Department of Molecular Oncology, BC Cancer Agency, 675 West 10th Avenue, Vancouver, BC, V5Z 1L3, Canada; Graduate Bioinformatics Training Program, University of British Columbia, 315- 2185 East Mall, Vancouver, BC, V6T 1Z4, Canada; Department of Statistics, University of British Columbia, 2207 Main Mall, Vancouver, BC, V6T 1Z4, Canada; Department of Molecular Oncology, BC Cancer Agency, 675 West 10th Avenue, Vancouver, BC, V5Z 1L3, Canada; Department of Pathology and Laboratory Medicine, University of British Columbia, 2211 Wesbrook Mall, Vancouver, BC, V6T 1Z7, Canada; Department of Computer Science, University of British Columbia, 2366 Main Mall, Vancouver, BC, V6T 1Z4, Canada

## Abstract

**Motivation:**

Cancer is driven by somatic mutations that result in the expansion of genomically distinct sub-populations of cells called clones. Identifying the clonal composition of tumours and understanding the evolutionary relationships between clones is a crucial task in cancer genomics. Bulk DNA sequencing is commonly used for studying the clonal composition of tumours, but it is challenging to infer the genetic relationship between different clones due to the mixture of different cell populations.

**Results:**

In this work, we introduce a new probabilistic model called PhyClone that can infer clonal phylogenies from bulk-sequencing data. We demonstrate the performance of PhyClone on simulated and real-world datasets and show that it outperforms previous methods in terms of accuracy and sample scalability.

**Availability and implementation:**

Source code is available on Github at: https://github.com/Roth-Lab/PhyClone under the GPL v3.0 license.

## 1 Introduction

Inferring the evolutionary history of cancer cell populations is a fundamental problem in cancer biology (Burrell *et al.* 2013, McGranahan and Swanton 2017). High throughput genomics methods have been transformative in this effort, providing insights into mechanisms of malignant ontogeny, therapeutic resistance and metastasis (Turajlic *et al.* 2019). While single-cell sequencing methods exist to precisely characterize tumour evolution (Zahn *et al.* 2017, Laks *et al.* 2019), their application has remained limited due to cost and technical challenges when applied to patient tumour samples. Thus, the field remains heavily reliant on bulk sequencing and computational deconvolution of tumours to infer clonal population structure (Dentro *et al.* 2021, Frankell *et al.* 2023).

Many tools for quantifying clonal populations in bulk sequencing have focused on clustering mutations that occur at the same cellular prevalence, i.e. occur in the same proportion of malignant cells (Miller *et al.* 2014, Roth *et al.* 2014, Deveau *et al.* 2018, Gillis and Roth 2020). These approaches assume that mutations with the same cellular prevalence across samples occur in the same set of cells. Clustering mutations based on cellular prevalence thus provides insight into which mutations originated during the same clonal expansions and have the same pattern of mutation loss (Gerstung *et al.* 2020). While effective for identifying clonal populations, clustering approaches ignore the underlying phylogenetic relationship among clones. To address this deficiency, a number of approaches have attempted to reconstruct the clonal phylogeny from bulk sequencing (Deshwar *et al.* 2015, Malikic *et al.* 2015, Popic *et al.* 2015, Wintersinger *et al.* 2022, Grigoriadis *et al.* 2024). Beyond providing an estimate of the phylogenetic tree, a key benefit of these approaches is that they allow for the genotypes and prevalence of clones to be resolved.

Methods for inferring clonal phylogenies from bulk sequencing generally make the infinite sites assumption (ISA), which implies that mutations with higher cellular prevalence cannot be acquired later than mutations with lower cellular prevalence (Dentro *et al.* 2017, McGranahan and Swanton 2017, Tarabichi *et al.* 2021). In the context of bulk sequencing this assumption can lead to ambiguities, and cannot, e.g. resolve whether two clonal populations are siblings or ancestor/descendants if the sum of cellular prevalence does not exceed one. This deficiency can partially be addressed by conducting multi-region sequencing (Tarabichi *et al.* 2021, Cortés-Ciriano *et al.* 2022), which has now become common practice in cancer evolution studies (Frankell *et al.* 2023). The key insight is that if a mutation has higher prevalence than another mutation in one sample, but the reverse relationship between the mutations is observed in a different sample, the mutations must originate in clones from different clades of the phylogeny, i.e. the associated clones lie on different branches (Tarabichi *et al.* 2021). In addition to only providing weak identifiability, the additivity assumption is violated if mutation loss occurs. In the context of cancer evolution, mutation loss can occur due to genomic instability leading to the deletion of large regions of the genome (McGranahan and Swanton 2017, Tarabichi *et al.* 2021). Frequent mutation loss has been identified in genomically unstable cancers in several studies (McPherson *et al.* 2016, Jamal-Hanjani *et al.* 2017, Gerstung *et al.* 2020, Watkins *et al.* 2020).

In this work, we develop a new method for inferring clonal phylogenies based on bulk sequencing. Given that bulk sequencing may only contain information to partially resolve the clonal phylogenetic structure, we adopt a Bayesian statistical approach, which allows us to infer a posterior distribution over the set of potential phylogenies compatible with the data. To address the fact that the number of clonal populations is unknown, we have developed a non-parametric Bayesian model of clusters related by a tree structure. A key benefit of our approach is the ability to marginalize the parameters associated with tree nodes (clonal prevalence), allowing us to perform posterior inference in the lower dimensional space of tree topologies. Inference in the collapsed space of discrete clustering trees still requires exploration of an exponential number of tree topologies with variable numbers of nodes. To address this issue, we developed an inference algorithm using particle Gibbs sampling (Andrieu *et al.* 2010) coupled with an auxiliary variable construction. In addition, our approach allows for partial information about clustering generated by fast non-phylogenetic methods to be incorporated. We demonstrate in the results, that these advances allow our approach to scale favourably in terms of running time and accuracy with increasing numbers of samples. In addition, we show how our proposed method can be adapted to allow for the potential of mutation loss for accurate inference in real datasets.

## 2 Materials and methods

**Figure 1.**
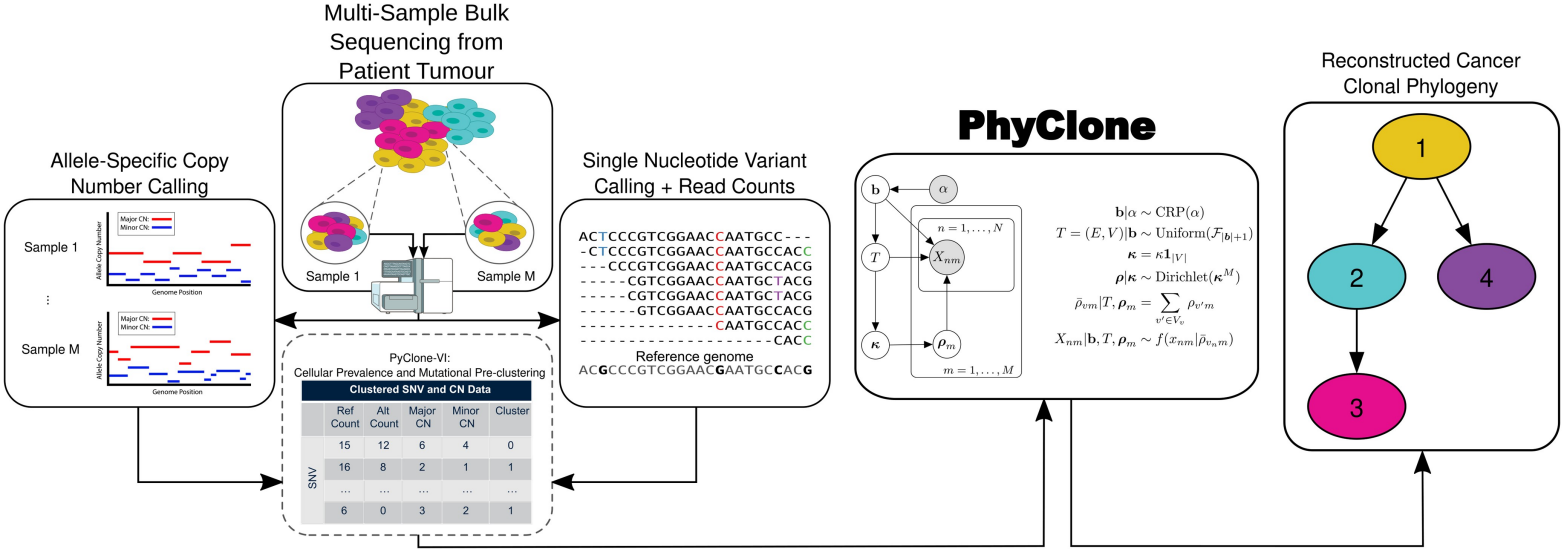
Overview of the PhyClone model. Outline of the high-level process of clonal phylogeny reconstruction from bulk sequencing used by PhyClone. As input PhyClone requires allele-specific read counts and copy number data for single nucleotide variants (SNVs) identified in one or more samples.

The PhyClone model takes as input: (i) allelic count data from somatic single nucleotide variants (SNVs); (ii) copy number variant (CNV) information; (iii) tumour content estimates from bulk sequence data of one or more related tumour samples (Fig. 1). Using this information PhyClone infers the posterior distribution of phylogenetic trees relating the clones in the sample. As part of this inference, we infer the number of clones, the sets of mutations that originate at each node in the tree, and the phylogenetic ordering of mutations. We marginalize prevalence of clones in each sample, but if needed, the prevalences can be reinstantiated via maximum *a posteriori* (MAP) estimation or sampling after tree inference.

We first describe the non-parametric Bayesian tree-structured clustering prior distribution used by PhyClone. We then provide a full description of the basic PhyClone model, and several extensions. Finally, we describe the inference strategy. More detailed discussion of the model and inference strategy are provided in the Supplementary Methods, available as supplementary data at *Bioinformatics* online.

### 2.1 Prior distribution over clone phylogenies

PhyClone employs the forest structured Chinese restaurant process (FS-CRP) as *a prior* over tree topologies and clusters. In this section, we briefly describe the generative process that defines the FS-CRP. In the first step of the FS-CRP, we partition SNVs into clusters assumed to have the same evolutionary history. To do so, SNVs are split into clusters via the Chinese restaurant process (CRP) (Aldous 1985, pg. 92). In the notation that follows, this partitioning of SNVs is represented by b, with b∈b representing an individual cluster in this partitioning.

In the second step of the FS-CRP, we sample a topology for the clonal phylogeny. A directed, rooted, forest of multifurcating arborescences (trees) with as many nodes as clusters is thus selected uniformly at random, and each cluster is associated to a node, ultimately forming a one-to-one relation between clusters and nodes. The sampled directed forest may exhibit multiple roots, so we use the observation that any rooted forest (i.e. any collection of rooted trees) over *K* nodes can be turned into a single-rooted tree over K+1 nodes by setting all root nodes to be children of a new empty root node.

In the third step, we assign clonal prevalence to each node. The clonal prevalence is defined to be the proportion of malignant cells that originate at a node, or equivalently the proportion of cells with the genotype defined by the union set of mutations assigned to nodes on the path to the root. To do so, for each individual sample provided, a vector of length equal to the number of nodes is sampled from a Dirichlet distribution. This enforces the constraint that the sum of all clonal prevalence within a sample adds to one. To obtain the cellular prevalence, i.e. the proportion of malignant cells that harbour an SNV, we move recursively up the tree from the leafs, summing the clonal prevalence of the node with the cellular prevalence of its children. This corresponds to the ISA which in turn implies that SNVs are propagated to all descendants from their clone of origin. The output of this process is a tree-structured clustering of SNVs, which we refer to as a clone phylogeny. In the clone phylogeny, each node has a collection of originating SNVs, clonal prevalence, and cellular prevalence defined.

### 2.2 PhyClone model

**Figure 2.**
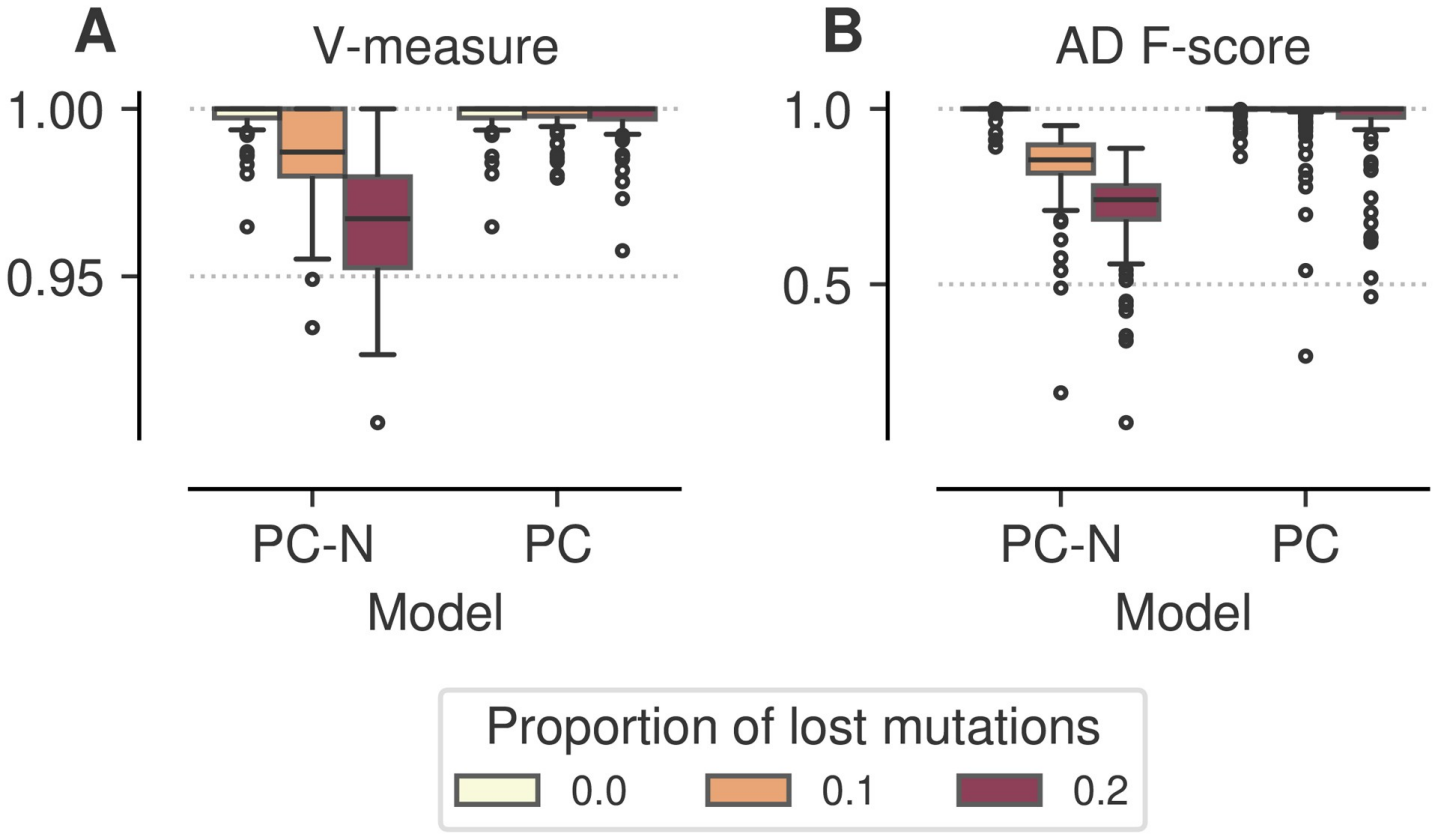
PhyClone performance with mutations loss. (A) V-measure and (B) ancestor–descendant *F*-score for PhyClone with (PC) and without outlier modelling (PC-N) as a function of the proportion of mutations lost.

Figure 1 outlines the PhyClone model for multiple samples with no outliers. For simplicity, in the following we will assume a single sample to keep the notation uncluttered (Fig. 1, available as supplementary data at *Bioinformatics* online). The observed data vector *X* contains the allelic read counts and copy number information for all mutations n∈[N]={1,…,N} used for computing the PyClone likelihood (Methods S1.1 and S1.2, available as supplementary data at *Bioinformatics* online). This model can be generalized to multi-region sequencing by sampling the clonal prevalence of each region from a Dirichlet distribution (Fig. 2, available as supplementary data at *Bioinformatics* online). To compute the cellular prevalence of a mutation, which will be used in the PyClone emission density for the allele counts of a mutation, let:

-
b denotes a clustering of the mutations, such that $b=\{b\subset \{1,\ldots ,N\}:b_{i}\cap b_{i^{\prime }}=\emptyset ,\cup b=\left[N\right]\}$.
  - More plainly, b is a set of SNV clusters *b*, such that the union of all clusters b∈b is equal to the set of all SNVs in *N*.
  - Furthermore, each cluster *b* in b is a unique set of SNVs, such that there is no overlap between any two clusters in b.
-
α denotes the concentration parameter used during clustering of b.
-
ρ denotes a vector of clonal prevalences sampled from a Dirichlet distribution of dimension |b|+1=|V|.
-
$T_{v}$ denotes the subtree of *T* rooted at node *v*.
-
$V_{v}$ denotes the set of nodes in $T_{v}$.
-
$C_{v}$ denotes the set of children nodes of *v*.
-
$v_{n}\in V$ denotes the node associated with cluster b∈b such that n∈b. That is, mutation *n* is in cluster *b*, and cluster *b* is assigned to tree node $v_{n}$.
-
${\bar{\rho }}_{v}=\sum_{v^{\prime }\in V_{v}}\rho_{v^{\prime }}=\rho_{v}+\sum_{v^{\prime }\in C_{v}}{\bar{\rho }}_{v^{\prime }}$

**Figure 3.**
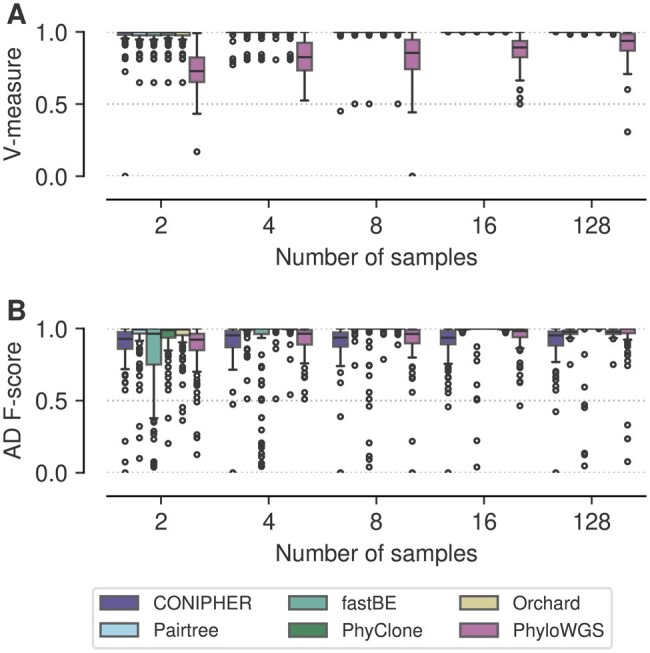
Performance on small TSSB dataset by number of samples. (A) V-measure, clustering accuracy. (B) Ancestor–descendant *F*-score.

In this notation, $\rho_{v}$ and ${\bar{\rho }}_{v}$ represent the clonal and cellular prevalence of node *v*, respectively. The likelihood is given by:


$$p(X|\rho ,b,T)=\prod_{n=1}^{N}f(x_{n}|{\bar{\rho }}_{v_{n}}),$$

where $f(x_{n}|{\bar{\rho }}_{v_{n}})$ represents the copy number and tumour content corrected likelihood of allele counts based on the PyClone model (Fig. 3, available as supplementary data at *Bioinformatics* online, Roth *et al.* 2014).

#### 2.2.1 Marginalizing clonal prevalence

For inference, it is beneficial to work with the collapsed joint distribution, where we marginalize the node parameters ρ. Let $\Delta_{k}$ denote the *k* simplex, $\Delta_{k}=\left\{\rho \in R_{+}^{k}:\sum \rho_{i}=1\right\}$.


$$\begin{array}{c} p(X,b,T)=\int_{\Delta_{\left|V\right|}}p(X,b,T,\rho )d\rho \\ =p(b|\alpha )p(T|b)\int_{\Delta_{\left|V\right|}}p(\rho |\kappa )p(X|\rho ,b,T)d\rho \\ =p(b|\alpha )p(T|b)\int_{\Delta_{\left|V\right|}}p(\rho |\kappa )\prod_{n=1}^{N}f(x_{n}|{\bar{\rho }}_{v_{n}})d\rho \end{array}$$

Computing $\int p(\rho |\kappa )\prod_{n=1}^{N}f(x_{n}|{\bar{\rho }}_{v_{n}})d\rho$ is non-trivial because of the dependence on the tree structure. We describe an efficient dynamic programming algorithm in the Supplementary Material (Section S1.5, available as supplementary data at *Bioinformatics* online) that computes the marginalized likelihood in $O(|V{|}^{2})$.

#### 2.2.2 Pre-clustering

For computational efficiency, PhyClone can use clustering information from fast non-phylogenetic approaches such as PyClone-VI (Gillis and Roth 2020). In principle, PhyClone can be used without pre-clustering. However, as it drastically increases the computational complexity, pre-clustering is recommended for WGS sized datasets.

Our approach enforces that any mutations clustered together by the pre-clustering step will be kept together. However, mutations assigned to different clusters by the pre-clustering algorithm can also be placed together by PhyClone; a feature, i.e. achieved through PhyClone’s ability to merge separate clusters (or single mutations for un-clustered inputs) together inside a node. This allows PhyClone to refine and improve upon the clustering based on the constraints imposed by the phylogeny.

#### 2.2.3 Outliers

To accommodate SNVs that do not satisfy the additivity assumption due to mutation loss, we allow the possibility of outliers. Each SNV is assigned *a prior* probability of being an outlier, $\nu_{n}$. We define a binary variable $o_{n}$ that indicates whether mutation *n* is an outlier. If a mutation is not an outlier, it joins a node in the tree and contributes to the likelihood as above. If the mutation is an outlier, it has the standard PyClone likelihood where we assume it has a cellular prevalence drawn from a Uniform distribution. We marginalize the cellular prevalence in this case using numerical integration.

The joint-likelihood with outliers becomes:


$$\begin{array}{c} p(X,b,T,o)=\prod_{n=1}^{N}[\nu_{n}\int_{0}^{1}f(x_{n}|\rho )d\rho {]}^{I(o_{n}=1)}\\ \times p(b|\alpha )p(T|b)\int_{\Delta_{\left|V\right|}}p(\rho |\kappa )\\ \times \prod_{n=1}^{N}[(1-\nu_{n})f(x_{n}|{\bar{\rho }}_{v_{n}}){]}^{I(o_{n}=0)}d\rho \end{array}$$

### 2.3 Inference

**Figure 4.**
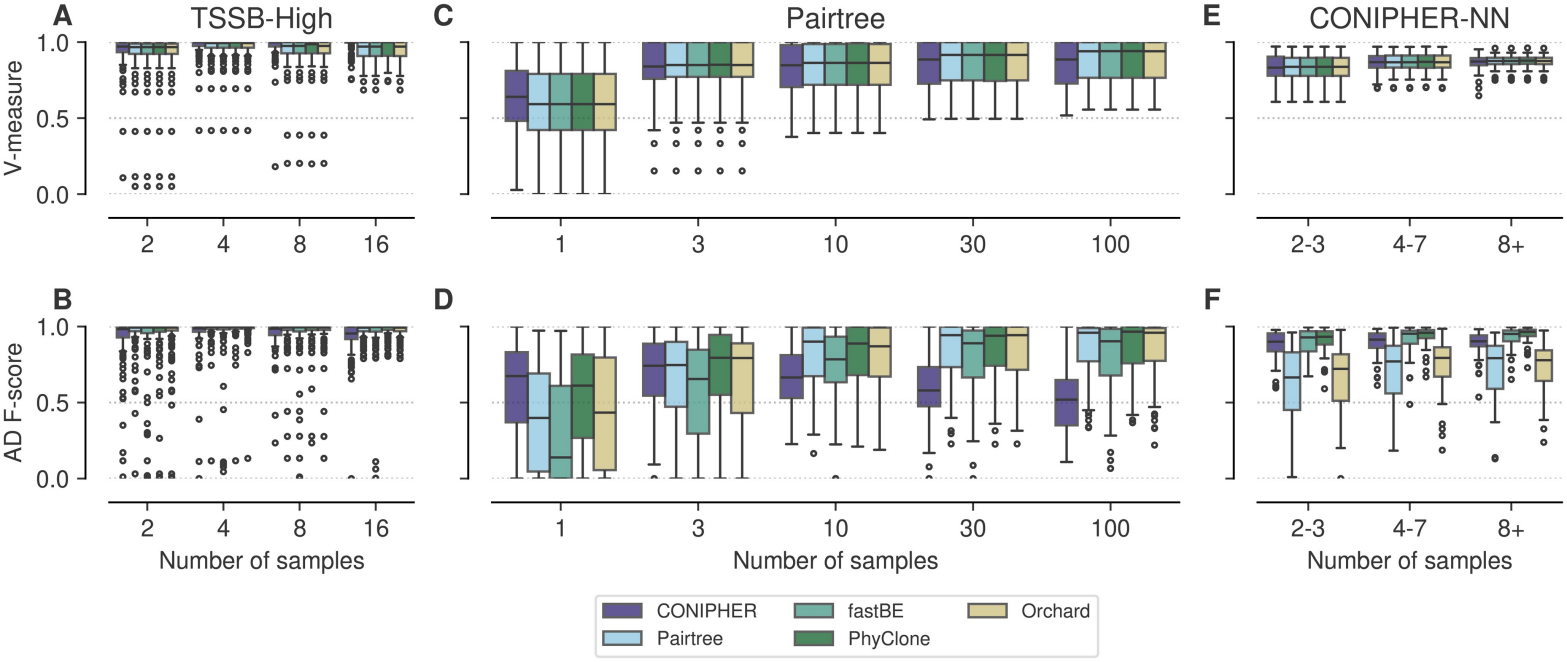
Performance on noise free synthetic data. V-measure and ancestor–descendant *F*-score for (A, B) high SNV TSSB (TSSB-High), (C, D) pre-clustered Pairtree, and (E, F) CONIPHER no-noise (CONIPHER-NN) datasets.

To simultaneously infer the posterior distribution of tree topologies and cluster assignments (*T* and b), we use a sequential Monte Carlo (SMC) algorithm. The SMC algorithm works by evolving a set of particles that are iteratively extended and reweighed (Fig. 4, available as supplementary data at *Bioinformatics* online), such that the final iteration yields an approximation of the posterior. The SMC design in PhyClone grows the rooted forest (the eventual phylogeny) from the bottom up. The primary motivation behind this bottom-up (or post-order) design is that it allows us to define a partial likelihood that, in unison with our dynamic programming marginalization algorithm, can be efficiently evaluated using the SMC approach.

We assume that a permutation, σ, of the mutation indices [*N*] has been given. At algorithmic time *t* of the SMC procedure we add data point $X_{\sigma (t)}=x_{t}$. The definition of the target density used to guide the SMC iterations can be found in Methods S1.6.1, available as supplementary data at *Bioinformatics* online.

A key parameter to define for SMC algorithms is the proposal function, $q(x_{t+1}|x_{t})$, which extends the state $x_{t}$ to a new state $x_{t+1}$. In the case of our algorithm, this corresponds to choosing which node in the tree to allocate mutation σ(t+1) to. To propose a new state at time t+1, we consider all possible states that can be reached by:

1. Adding a data point to an existing root node.
2. Creating a new node choosing a possibly empty subset of children from the existing root nodes as children.

We randomly choose whether to join an existing root node or start a new node. When adding a mutation to an existing root node, we compute the probabilities of the resulting trees with the mutation attached at each root node. When adding a mutation to a new node, we randomly sample the set of children. This approach provides a compromise between the quality of trees proposed and the computational complexity.

#### 2.3.1 Overcoming the impact of data ordering

**Figure 5.**
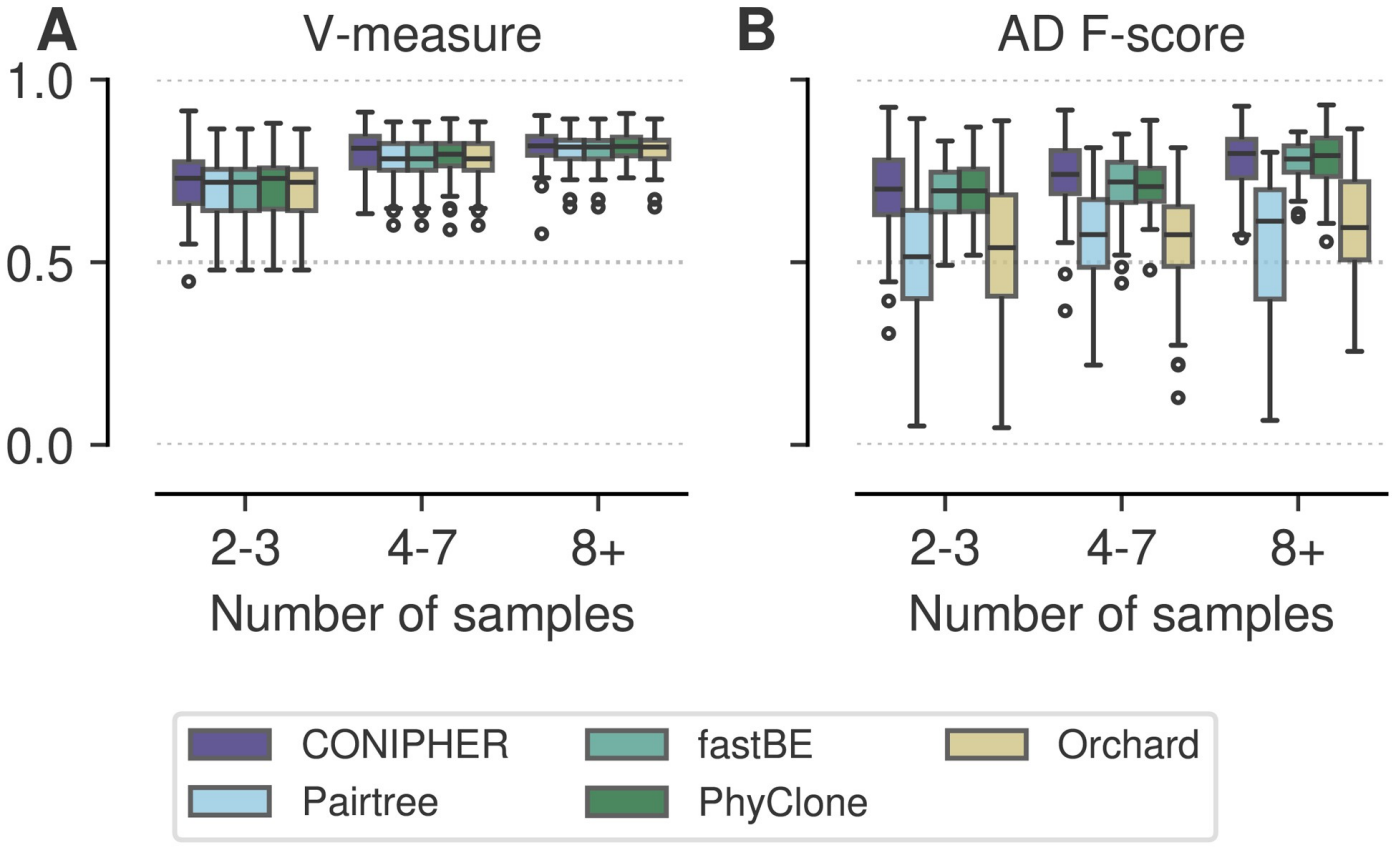
Performance on CONIPHER noise dataset. (A) V-measure and (B) ancestor–descendant *F*-score for methods on the CONIPHER noise dataset.

**Figure 6.**
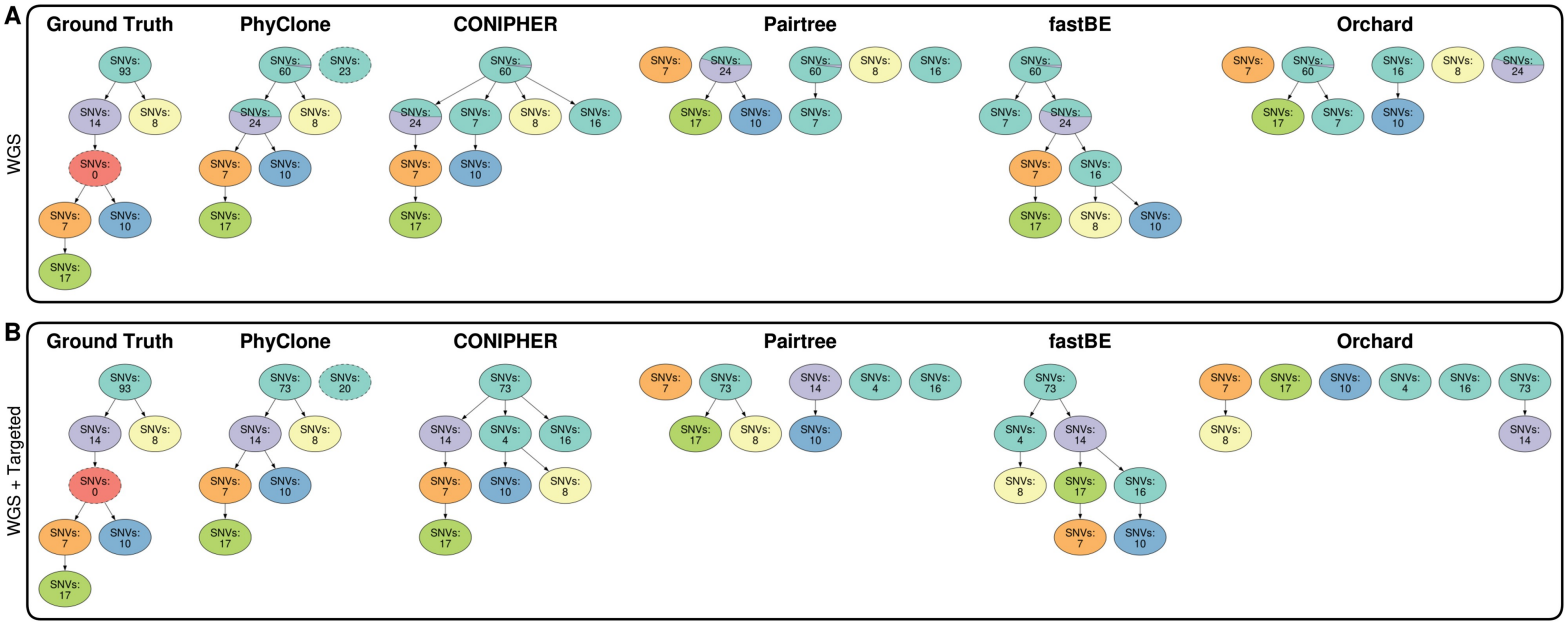
HGSOC patient 3 clonal phylogenetic trees. (A) Predicted trees built using purely whole-genome sequencing (WGS) data. (B) Predicted trees built using a combination of WGS and targeted deep sequencing data (McPherson *et al.* 2016). From left to right: ground-truth phylogenetic tree inferred from single-cell and targeted deep sequencing data (McPherson *et al.* 2016), nodes with a dashed border denote clones that are defined only by the absence of SNVs from the parent. Predicted trees built using: PhyClone with outlier modelling; CONIPHER; Pairtree; fastBE; and Orchard. Colours of nodes in method inferred trees correspond to the single nucleotide variant clonal (SNV) assignment from ground truth. Predicted tree nodes with a dashed border denote SNVs that the method has classified as outliers/lost.

A limitation of the procedure so far described is that not all trees can be sampled due to the fixed order of the data points. Specifically, if $x=\sigma_{i}$ and $y=\sigma_{j}$, where i<j then any tree where *x* is an ancestor of *y* cannot be sampled given a fixed σ (Figs 5–7, available as supplementary data at *Bioinformatics* online). For example, the first element of σ can never be a root node unless the sampled tree has only a single node.

To address this issue we use a particle Gibbs (PG) sampler to embed our SMC procedure in a more general Markov chain Monte Carlo sampler (Andrieu *et al.* 2010). At a high level, we treat σ as an auxiliary variable and alternate between sampling σ and (T,b). Sampling of σ is constrained such that the SMC procedure could generate the current configuration of *T* and b (Fig. 8, available as supplementary data at *Bioinformatics* online).

To see that this algorithm yields a sampler that can sample all trees, note that the single node tree can be reached at any iteration of the algorithm. From this configuration, the sampling σ is uniform, thus allowing any ordering of mutations to be chosen for the subsequent update of (T,b).

#### 2.3.2 Additional MCMC moves

To improve the mixing of the sampler, it is beneficial to include additional moves in addition to the particle Gibbs updates. We use two moves, neither of which change the number of nodes in the tree.

The first move is a subtree prune-regraft (Swofford and Sullivan 2009, pgs. 283, 286). To perform this move, we select a node at random from the tree and remove the subtree rooted at this node. We then compute the probability of the tree which would result from attaching the subtree to a node in the tree. We do this for all nodes in the tree and Gibbs sample a new attachment node.

The second move we consider is to sample new node assignments $v_{n}\in V$ for each data point $x_{n}\in X$ without changing the topology. To do this, we first shuffle the data point ordering σ, obtaining a new permutation of the indices [*N*], $\sigma^{\prime }$. Then, for each datapoint $x_{i}\in X_{\sigma^{\prime }}$, if removing $x_{i}$ from its current node $v_{i}$ does not empty $v_{i}$, we compute the probability of each tree resulting from having added $x_{i}$ to $\forall v_{i^{\prime }}\in V$, such that $v_{i^{\prime }}\neq v_{i}$. We then sample a new candidate tree from this set of topologically-stable (i.e. isomorphic) yet data-clustering perturbed configurations.

### 2.4 Experiments

We tested PhyClone against five state-of-the-art methods: PhyloWGS (Deshwar *et al.* 2015), Pairtree (Wintersinger *et al.* 2022), CONIPHER, Orchard (Kulman *et al.* 2024), and fastBE (Schmidt and Raphael 2024). Here, we describe the datasets, program settings, and performance metrics used for these benchmarking experiments (see Table 1).

**Table 1.** Method feature comparison table.

Method	Publication year	Infers mutational loss	Sequencing error correction	Clusters mutations	Computes clonal prevalence	Multiple solutions
PhyloWGS (Deshwar *et al.* 2015)	2015	Yes	No	Yes	No	Yes
Pairtree (Wintersinger *et al.* 2022)	2022	No	Yes^a^	No^b^	Yes	Yes
CONIPHER (Grigoriadis *et al.* 2024)	2023	Yes	Yes	Yes	Yes	Yes
Orchard (Kulman *et al.* 2024)	2024	No	Yes^a^	No^b^	Yes	Yes
fastBE (Schmidt and Raphael 2024)	2024	No	No	No^b^	No	No
PhyClone	2025	Yes	Yes	Yes	Yes	Yes

a Method cannot correct for sequencing error or mutational loss during tree building, but an included pre-processing script can be used to adjust the input prior to tree building.

b Method does not cluster mutations during tree building but can do so separately.

#### 2.4.1 Datasets

A combination of newly simulated synthetic datasets, previously published synthetic datasets, and real human cancer data were used to perform benchmarking of the methods.

##### 2.4.1.1 FS-CRP loss data

Tree topologies and clusterings were simulated using the FS-CRP prior. Mutational loss was simulated by first randomly selecting a set of mutations equal to the proportion to be lost; e.g. if there were 100 mutations in total and the loss proportion was 0.2, we would randomly select 20 of those mutations to be lost. A node is then selected from which the set of mutations is to be ‘lost’ from; the loss-node is selected such that it is a descendant node from the node that the lost mutations originate from. All mutations that are members of the lost set are given positions on the same chromosome, to simulate the genomic locality of sub-clonal chromosomal region loss. All nodes downstream of (and including) the loss-node then have the cellular prevalence of the lost set of mutations subtracted from the node’s total, to reflect the impact of having lost the mutations.

We simulated 100 trials for each level of mutation proportion to lose, with the mutation proportions being {0.0,0.1,0.2}. Each trial simulated 600 SNVs, with a depth of 1000, eight samples, and a tumour content of 1.0.

##### 2.4.1.2 Tree-structured stick breaking data

Tree topologies and clusterings were simulated using the tree-structured stick breaking (TSSB) prior. The TSSB prior is an alternative non-parametric Bayesian prior over clusters and tree topologies (Ghahramani *et al.* 2010). Allelic count data were simulated as diploid heterozygous mutations with a tumour content of 1.0. Two TSSB datasets were produced. Both datasets that were simulated had a read depth of 1000, sample number levels of [2, 4, 8, 16], and tumour content of 1.0. The small dataset (TSSB-Low) was simulated with 100 SNVs and the larger (TSSB-High) had 10 000 SNVs. Additionally, the small TSSB dataset also included runs with 128 samples.

##### 2.4.1.3 Pairtree data

We used previously published data from Wintersinger *et al.* (2022). In order to avoid clashing with the notation previously established in this article, the variables describing the experimental parameters below have been redefined from those used in the Pairtree publication; however, their values and meaning remain unchanged. The Pairtree data consisted of simulated datasets that varied according to combinations of the following parameters: number of sub-clones, K={3,10,30,100}; number of samples, M={1,3,10,30,100}; total depth, D={50,200,1000}; and number of mutations equal to dataset K*N, where N={10,20,100}; with four replicates each, for a total of 576 datasets.

##### 2.4.1.4 CONIPHER data

We used previously published data from Grigoriadis *et al*. We used datasets 1 and 2, each with a total of 150 trials, which we refer to as the ‘noise’ and ‘no-noise’ datasets, respectively. For analysis, we split each dataset into three categories according to number of samples: 2–3, 4–7, and 8+.

##### 2.4.1.5 High grade serous ovarian cancer data

We used data from a previous single-cell study of high grade serous ovarian cancer (HGSOC) where ground-truth trees were inferred from single-cell, whole-genome, and targeted deep sequencing data (McPherson *et al.* 2016). Two sets of data were reconstructed for the HGSOC experiments; one dataset consisted of purely whole-genome sequencing (WGS) bulk data, while the second dataset replaced the WGS allele counts with the targeted deep sequencing counts for the SNVs which were present in both sets; these datasets are referred to as WGS and WGS + Targeted. Pre-processing involved reconstruction of copy number, read count, and cluster assignment data from the supplementary tables, available as supplementary data at *Bioinformatics* online, provided with the original study.

Methods were provided with the complete set of processed and clustered SNVs (per patient/dataset) on which to infer their clonal phylogeny solutions. However, the ground-truth trees established by McPherson *et al.* (2016) were defined only by the subset of mutations that were selected for single-cell targeted deep sequencing. Thus, when computing metrics, each method’s phylogeny solution was post-processed to contain only the ground-truth defining mutations, with any resulting empty nodes from this process being collapsed into their parent node so as to not corrupt the inferred topology.

#### 2.4.2 Program parameters

Pre-clustering was performed for synthetic data (2.4.1, 2.4.1, and 2.4.1) using PyClone-VI v0.1.6 initialized with 100 clusters, 100 random restarts, and using the binomial allele-count density. For the HGSOC data (2.4.1), we used the clusters provided in the original publication. Pre-clustered results were supplied to CONIPHER, Pairtree, Orchard, fastBE, and PhyClone for all experiments. Each method was allowed 48 h wall-clock time to complete each individual trial in each dataset.

PhyClone (version 0.7.0) was run with the following parameters: four independent chains, 100 burn-in iterations, 5000 MCMC iterations, 100 particles, Beta-Binomial allele-count density, and semi-adapted proposal kernel; furthermore, for the version of PhyClone that includes outlier modelling, we set the ‘–assign-loss-prob’ flag and accompanying (default) outlier prior probabilities of 0.0001 and 0.4 for the low and high loss prior probabilities, respectively. The final output tree for PhyClone was selected by MAP joint-likelihood.

PhyloWGS, Pairtree, CONIPHER, Orchard, and fastBE methods were run with default parameters, with the exception of CONIPHER in the HGSOC experiments, where the ‘min_cluster_size’ option was set to 3. This setting was adjusted in the HGSOC experiments to allow for CONIPHER to potentially infer the loss of smaller mutation clusters, a situation which was present in the ground truth.

For all point-estimate (i.e. single tree) metrics, the default or top-scoring tree provided by each method was used for the evaluation.

#### 2.4.3 Metrics

Each experimental method was measured against ground truth in order to assess the accuracy of clonal phylogeny reconstruction and SNV assignments. The following metrics were employed:

- V-measure: a metric for measuring clustering accuracy (Rosenberg and Hirschberg 2007). That is, this metric is concerned with quantifying how well a method clusters together mutations that are clustered in the ground truth, while excluding those that are not.
- Ancestor–descendant *F*-score (AD *F*-score): a reconstruction accuracy metric which measures the *F*-score (or *F*1-score) of a method’s phylogenetic reconstruction from the perspective of SNV ancestor–descendant relationships (Fig. 9, available as supplementary data at *Bioinformatics* online). Positive cases result when an SNV is assigned to a node that can form an upward path to the node where a corresponding true ancestral SNV is assigned.
  - Put more simply, given a tree, for a given node, and for each mutation in the node: this metric constructs a set of ancestor–descendant pairs from the current mutation to all mutations present in the descendant nodes. This set of pairs is then compared against the set of true ancestor–descendant pairs from the ground-truth, and from these two sets of pairs, true and false positives and negatives are tabulated. These measures are then used to compute the *F*-score.
  - This metric is meant to evaluate a method’s ability to accurately reconstruct pair-wise ancestral relationships between mutations.
- Posterior metrics: the following metrics are measured over the set of all unique solutions produced by a method, and are each computed as likelihood-weighted averages.
  - Log perplexity ratio (LPR): also known as the VAF reconstruction loss, this metric measures the LPR between the clonal cellular prevalence matrices returned by a method against that of the ground truth. To interpret this metric, a lower (including negative values) score is better (Wintersinger *et al.* 2022, Kulman *et al.* 2024). To not bias the results of the LPR, all method solutions were scored using the PhyClone marginalization algorithm.
  - Relationship reconstruction error (RRE): following an exhaustive construction of all possible valid topologies (i.e. those that do not violate the ISA) that fit the ground-truth clonal cellular prevalence matrix, this metric evaluates how well a method’s solutions have reconstructed the pairwise evolutionary relationships present in the set of valid ground-truth topologies (Wintersinger *et al.* 2022, Kulman *et al.* 2024).

We only report the posterior metrics for the TSSB and Pairtree datasets, as the CONIPHER dataset does not comply with the perfect phylogeny assumption; the tree enumeration used by the RRE metric fails in this case.

Significance testing for differences in performance for each benchmarking experiment was performed via Friedman–Nemenyi testing. If the metric being tested demonstrated a global significance difference (*P*-value <.01) as per the Friedman test, a pairwise *post hoc* Nemenyi test was then run to assess which methods performed significantly (*P*-value <.01) differently from each other. Subsequently, the mean difference was used to determine the direction of said significance (i.e. which performed better out of the pair) (Demšar 2006).

## 3 Results

### 3.1 Outlier modelling mitigates major additivity violations

While the PyClone emission model can account for the effect of sequencing error (Roth *et al.* 2014), it cannot correct for larger violations of the additivity assumption. Thus, the deletion of a large number of SNVs due to copy number variation could conceivably pose a challenge.

To investigate this, we first simulated data from the PhyClone model allowing for mutation loss (data described in Section 2.4.1). We used this dataset to assess the performance of including an outlier state in the PhyClone model. As this dataset is inherently biased to favour PhyClone, we omit other methods from this comparison. The results of this experiment suggest that the performance of the outlier naïve PhyClone model (PC-N) deteriorates as the proportion of mutations lost in the tree increases (Fig. 2). However, the addition of the outlier state addresses this issue, making the method far more robust to loss. In addition, the inclusion of outlier modelling does not appear to impact performance when no mutation loss is observed (Fig. 2).

In sequel, we only consider the PhyClone model with outlier modelling in the experiments, as we expect that allowing for mutation loss will be the default use-case when analysing real data.

### 3.2 Scalable and accurate Bayesian inference

We first analysed the small TSSB dataset with 100 SNVs. We note, this model is equivalent to the PhyloWGS model and thus should favour that method. Our goal was to compare the accuracy and efficiency of different modelling and inference approaches when data is generated from a well-defined statistical model. PhyClone and PhyloWGS are fully Bayesian models, whereas Pairtree uses a Gaussian approximation to perform MAP estimation, CONIPHER optimizes a heuristic objective, fastBE employs a two stage regression and optimization strategy, and Orchard implements a factorized approximation of the posterior over phylogenetic space. In addition, PhyClone, Pairtree, CONIPHER, Orchard, and fastBE are all capable of utilizing pre-clustering of the data to scale inference.

The clustering and tree accuracy results for this experiment are summarized in Fig. 3, while the posterior metrics can be viewed in Fig. 10C and D, available as supplementary data at *Bioinformatics* online. For the TSSB-Low dataset, PhyClone significantly outperformed all other methods in both RRE and LPR. PhyClone also significantly outperformed all other methods in AD *F*-score, while additionally significantly outperforming PhyloWGS in V-measure. Furthermore, no method was found to significantly outperform PhyClone in any of the aforementioned metrics. PhyloWGS was significantly slower than all other methods, requiring over an order of magnitude more time to finish than other approaches, with the gap widening with larger numbers of samples (Fig. 11, available as supplementary data at *Bioinformatics* online).

This experiment supports the idea that fully Bayesian inference can be more accurate. Furthermore, pre-clustering of data can accelerate inference without compromising accuracy. Based on the long running times observed for PhyloWGS, and the fact performance was not significantly better than other approaches under conditions which favour it, we did not include it in subsequent experiments.

### 3.3 Robust performance across phylogenetic models

We next sought to compare the performance of the methods across a range of clonal phylogeny simulation strategies. We analysed the large TSSB (TSSB-High), pre-clustered Pairtree, and CONIPHER no-noise (CONIPHER-NN) datasets. Datasets spanned a range of copy number, sample number, mutation count, and tumour content values. All data in these experiments were simulated to satisfy the ISA. Results for this experiment are summarized in Fig. 4 and Fig. 10, available as supplementary data at *Bioinformatics* online.

For AD *F*-score, PhyClone significantly outperformed both Orchard and Pairtree regardless of dataset; while in terms of V-measure, PhyClone significantly outperformed Pairtree and Orchard in the TSSB-High dataset. The performance advantages of PhyClone over CONIPHER were significant for both metrics in all cases except for V-measure on the TSSB-High dataset. PhyClone significantly outperformed fastBE in AD *F*-score across the pre-clustered Pairtree data.

PhyClone was only significantly outperformed in V-measure for the TSSB-High dataset, and only by one method, CONIPHER. In no case, across all datasets, did any method significantly outperform PhyClone in AD *F*-score.

Posterior metrics for the pre-clustered Pairtree and TSSB-High datasets are summarized in Fig. 10, available as supplementary data at *Bioinformatics* online. For the pre-clustered Pairtree dataset, PhyClone significantly outperformed both CONIPHER and fastBE in RRE, additionally, PhyClone significantly outperformed CONIPHER, fastBE, and Pairtree in LPR. In the posterior metrics measurement of the TSSB-High dataset, PhyClone was found to significantly outperform all other methods in LPR, while significantly outperforming Pairtree, fastBE, and Orchard in RRE. In terms of the posterior metrics LPR and RRE, no method was found to significantly outperform PhyClone.

For the pre-clustered Pairtree dataset, we also explored how the performance of methods varied as a function of the number of nodes in the tree, as opposed to the number of samples (Fig. 12, available as supplementary data at *Bioinformatics* online). In general, the performance of all methods deteriorated as the number of nodes increased. However, the relative performance rankings remained similar as a function of the number of nodes. As we show in Fig. 13, available as supplementary data at *Bioinformatics* online, the performance degradation is related to the ratio between the number of tree nodes and the number of samples for all methods. In general, this result would suggest one should not expect to resolve significantly more clones than the number of samples acquired when doing bulk sequencing.

Overall, these experiments suggest that PhyClone is relatively robust to the phylogenetic data generation model. Furthermore, PhyClone tends to benefit more than other approaches from increasing numbers of samples. This is reflected in both increasing median performance, and reduction in interquartile range of results for V-measure, AD *F*-score, and posterior metrics.

### 3.4 Accurate inference in the presence of noise

We next considered the impact of ‘noise’, i.e. mutations which fail to satisfy ISA. We analysed a previously published dataset, which we refer to as the CONIPHER noise dataset, which includes clusters of mutations that do not satisfy the ISA due to sequencing error or mutation loss. The results of this experiment are summarized in Fig. 5.

PhyClone, CONIPHER, and fastBE had no significant performance differences, and all three methods significantly outperformed Pairtree and Orchard in terms of AD *F*-score. As in the previous experiments, no method was found to significantly outperform PhyClone. Again, PhyClone benefited the most from increasing numbers of samples, with both better median performance and reduced variability.

Additionally, an experiment was run on a copy number perturbed version of the CONIPHER-NN dataset, the results of which are summarized in Fig. 14, available as supplementary data at *Bioinformatics* online. Across the copy number perturbed dataset, no method significantly outperformed PhyClone in any metric. However, PhyClone significantly outperformed fastBE, Orchard, and Pairtree for both AD *F*-score and V-measure, while significantly outperforming CONIPHER in AD *F*-score. Notably, the impact of increasing copy number error in this experiment appears to be minimal across the methods. We believe this effect is seen due to the PyClone-VI pre-clustering step used by all methods, which is somewhat robust to sample specific copy number errors. This is likely due to the fact the PyClone emission model allows for uncertainty over mutational genotype, as opposed to clustering based on a point-estimate of cellular prevalence.

### 3.5 PhyClone allows for accurate inference of clone phylogenies with mutation loss

To assess how mutation loss impacts the performance of all methods, we analysed previously published data from three patients with HGSOC (McPherson *et al.* 2016). Two patients (patients 2 and 3) exhibited complex forms of mutation loss, where clones experienced the loss of a subset of ancestral mutations. These results were validated with targeted single-cell sequencing, which we used as ground truth.

Pairtree struggled to resolve valid tree topologies (i.e. a single-rooted directed acyclic graph) across all three patients included in this dataset, as such this section focuses on the results provided by PhyClone, CONIPHER, Orchard, and fastBE.

PhyClone, CONIPHER, and fastBE all appeared to capably reconstruct the phylogenetic history of the clones in patients 2 and 9, with varying levels of resolution; while Orchard was only able to produce an accurate reconstruction for patient 9, and only when provided with the WGS with targeted deep sequencing counts (WGS + Targeted), Figs 15 and 16, Table 1, available as supplementary data at *Bioinformatics* online. For patient 3, in both forms of the data, WGS (Fig. 6A) and WGS with targeted deep sequencing counts (Fig. 6B), PhyClone was able to correctly identify and remove two SNV clusters that were found to be lost in ground-truth clones below the root. Neither CONIPHER nor fastBE were able to remove the lost SNVs and thus were unable to completely reconstruct the ground-truth phylogeny. The impact of the correct removal of the lost SNVs is further reflected in the performance metrics where PhyClone outperforms all methods for patient 3 (Table 1, available as supplementary data at *Bioinformatics* online).

## 4 Discussion

In this work, we have presented PhyClone, a new statistical method for reconstructing clonal phylogenies from multi-sample bulk-sequencing data. PhyClone is based on a Bayesian non-parametric model, the FS-CRP, which we develop in this work. A key feature of using the FS-CRP in PhyClone is the ability to marginalize the clonal prevalence given the constraints imposed by the tree. This allows us to perform collapsed inference in a parameter space that does not depend on the number of samples. As we demonstrate in the results, this improves the efficiency of sampling and accuracy of results as additional samples are used. In addition, we have developed a Particle Gibbs sampler for efficient inference. A key innovation of this inference scheme is an auxiliary variable construction over the sequence of points visited during each sequential Monte Carlo round.

The key limitation of PhyClone is the underlying PyClone model for correcting for mutational genotype. In particular, the model is not capable of modelling regions of sub-clonal copy number variation. However, as the number of samples increases we expect this deficiency to be less acute. For example, the HGSOC dataset analysed has numerous sub-clonal copy number events (McPherson *et al.* 2016, 2017). In future, the PyClone model could also be improved to adjust for sub-clonal copy number variation following approaches such as those in Frankell *et al.* (2023).

PhyClone presents a robust and scalable Bayesian method for clonal history reconstruction from multi-sample bulk-sequencing cancer data. Regardless of the source of synthetic data, PhyClone performed as well or frequently better than other state-of-the-art methods. Furthermore, PhyClone was able to reconstruct accurate phylogenies in real datasets in the presence of mutation loss. With its ability to leverage pre-clustering information, PhyClone easily scales to whole-genome scale datasets. PhyClone also scales well in the number of samples analysed in terms of running time and accuracy, allowing for extensive multi-region sequencing datasets to be analysed.

## Supplementary Material

btaf344_Supplementary_Data

## Data Availability

Pairtree (Wintersinger *et al.* 2022), CONIPHER (Grigoriadis *et al.* 2024), and HGSOC (McPherson *et al.* 2016) datasets are available from their respective publications. Pairtree datasets were retrieved from https://github.com/morrislab/pairtree-experiments and CONIPHER datasets were retrieved from Grigoriadis *et al.* 2024. TSSB synthetic datasets, FS-CRP loss synthetic datasets, method input files, and method output files for all experiments are available from Zenodo, https://doi.org/10.5281/zenodo.13240565.

## Code availability

PhyClone source code is available on Github at: https://github.com/Roth-Lab/PhyClone. An archival snapshot of the source code for the version of PhyClone used during benchmarking is available from Zenodo, https://doi.org/10.5281/zenodo.15062184.
