# Supplementary material for: PhyClone: accurate Bayesian reconstruction of cancer phylogenies from bulk sequencing
Source: Bioinformatics. 2025 Jun 13;41(7):btaf344. doi: 10.1093/bioinformatics/btaf344 (PMC12964358; doi:10.1093/bioinformatics/btaf344)
Supplement: btaf344_Supplementary_Data [file btaf344_supplementary_data.zip › supp.pdf]

S1 Supplementary Methods

S1.1 Allele count mode

PhyClone uses the same approach for correcting mutational genotype and tumour content as PyClone [3]. In this section we will review this approach mainly to establish notation. The data for the model is allele counts from N mutations from S samples. For simplicity, we will suppress the index n for the mutation and s for the sample in this section. We will assume that each mutation divides the set of cells that were sequenced into three sub-populations (Figure S3).

1. The normal cell population consisting of cells with healthy germline genomes.
2. The reference cell population which consists of cancer cells without the mutation in question.
3. The variant cell population which consists of cancer cells with the mutation in question.

Let $\mathcal{G} = (A, B, AA, AB, \cdots)$ be the set of all genotype where A and B represent reference and variant alleles respectively. For example AB would represent a heterozygous variant with total copy number 2. We assume the genotype of all cells within each sub-population is constant. Let $\mathbf{G} = (G_N, G_R, G_V) \in \mathcal{G}^3$ be a vector where the entries are the genotype of the normal, reference and variant populations respectively. Let t be the proportion of cancer cells in the sample. This is often referred to as the tumour content, tumour purity or cellularity of the sample. Let $\bar{\rho}$ denote the cellular prevalence of a mutation, that is the proportion of cancer cells with the mutation or equivalently the relative proportion of cancer cells in the variant population. This is often referred to as the cancer cell fraction (CCF) or cellular prevalence of the mutation. In sequel we will use tumour content and cellular prevalence.

Let ϵ be the assumed sequencing error rate. Let:

1. $a(G) : \mathcal{G} \to \mathbb{N}$, $b(G) : \mathcal{G} \to \mathbb{N}$ be functions which map a genotype to the number of A and B alleles respectively.
2. $c(G) : \mathcal{G} \to \mathbb{N}$ be defined as $c(G) = a(G) + b(G)$ which is the total copy number of the loci.
3. $\mu(G) : \mathcal{G} \to \mathbb{N}$ be defined as:

$$\mu(G) = \min\left\{\max\left\{\frac{b(G)}{c(G)}, \epsilon\right\}, 1-\epsilon\right\}.$$

   Which can be interpreted as the probability of sampling a read with the mutation from a population with genotype G.

Let $\xi(\mathbf{G}, \bar{\rho}, t)$ be the probability of sampling a read with the variant allele. We assume that we have an infinite initial population of cells which are sampled when sequencing. With this assumption the probability of sampling a read with a variant allele is roughly proportional to the number of copies of the variant allele in the input pool of DNA.

More formally, accounting for sequencing error, the probability of sampling a variant allele is given by the following equation.

$$\begin{aligned}
\xi(\mathbf{G}, \bar{\rho}, t) &= \frac{1}{Z}(1-t)c(G_N)\mu(G_N) \\
&+ \frac{1}{Z}t(1-\bar{\rho})c(G_R)\mu(G_R) \\
&+ \frac{1}{Z}t\bar{\rho}c(G_V)\mu(G_V) \\
Z &= (1-t)c(G_N) + t(1-\bar{\rho})c(G_R) + t\bar{\rho}c(G_V).
\end{aligned}$$

Now we observe d total reads covering the mutation in the sample, of which x contain the mutant allele. Thus

$$p(x|d, \boldsymbol{G}, \bar{\rho}, t) = \text{Binomial}(x|d, \xi(\mathbf{G}, \bar{\rho}, t))$$

If the data has more variance than can be explained by a Binomial model we can instead use:

$$p(x|d, \boldsymbol{G}, \bar{\rho}, t, \gamma) = \text{BetaBinomial}(x|d, \xi(\mathbf{G}, \bar{\rho}, t), \gamma).$$

Where the Beta-binomial distribution is parameterised by the mean $\xi(\mathbf{G}, \bar{\rho}, t)$ and precision (inverse of variance) γ.

So far we have assumed the genotypes of the sub-populations were known. In general this is not true for the reference and the variant populations. Instead it is typical to observe allele specific copy number estimates for the region overlapping a mutation. Using this information we can elicit a prior over a set of plausible genotypes. We explain how to do this in the next section. For now assume we have a vector $\boldsymbol{\pi}$ of prior probabilities where π_i is the prior probability of the ith plausible joint genotype, $\mathbf{G}_i$, of the populations. We can write to the probability of the observed data marginalising over all plausible genotypes as follows.

$$p(x|d, \boldsymbol{\pi}, \bar{\rho}, t) = \sum_i \pi_i \text{Binomial}(x|d, \xi(\mathbf{G}_i, \bar{\rho}, t))$$

or

$$p(x|d, \boldsymbol{\pi}, \bar{\rho}, t, \gamma) = \sum_i \pi_i \text{BetaBinomial}(x|d, \xi(\mathbf{G}_i, \bar{\rho}, t), \gamma).$$

We will call this the PyClone$(x|d, \boldsymbol{\pi}, \bar{\rho}, t)$ distribution in sequel where with some abuse of notation we ignore whether a Binomial or Beta-Binomial distribution is used.

We note that if we define the clonal prevalence of a mutation to be the proportion of cancer cells from a clonal population. Then the cellular prevalence is the sum of clonal prevalence for all clonal populations which have the mutation. In what follows the clonal prevalence will be the primary quantity of interest, though the cellular prevalence is required for the allele count model.

S1.2 Eliciting mutational genotype priors

Let c_{major} and c_{minor} denote the major and minor allele copy number for the region overlapping the mutation in the sample. We will use the "major copy number" method for setting potential genotype priors. This method considers two cases. In the first case, the mutation occurs before the copy number event. In this case the reference population genotype matches the normal population. We consider all possible mutational genotypes for the variant population with up to c_{major} chromosomes containing the variant. In the second case, the mutation occurs after the copy number event. In this case the reference population has $c_{major} + c_{minor}$ reference alleles. The variant population has 1 variant allele and $c_{major} + c_{minor} - 1$ reference allele. We set the prior weights to be equal for all possible mutational genotypes. For example suppose we have that $c_{major} = 2$ and $c_{minor} = 1$ and the normal copy number is 2. We have the following possible genotypes

- $\mathbf{G}_1 = (AA, AA, AAB)$
- $\mathbf{G}_2 = (AA, AA, ABB)$
- $\mathbf{G}_3 = (AA, AAA, AAB)$

each with prior probability $\frac{1}{3}$.

Note that if allele specific copy number is not available then c_{major} can be set to the total copy number and c_{minor} to zero.

S1.3 Generative model

In what follows we let $F = (E, V)$ denote a rooted forest, that is a graph with a directed edge set E and vertices set V. A root node is defined to be a vertex in V with incoming degree zero. A rooted tree, denoted by $T = (E, V)$, is a rooted forest with one root node. We define $\mathcal{F}$ to be the space of all rooted forests, and $\mathcal{F}_k = \{F = (E, V) \in \mathcal{F} : |V| = k\}$ to be the set of all forests with k nodes. We let $\mathbf{b} = \{b \subset \{1, \ldots, N\} : \bigsqcup b = [N]\}$ denote a partition of the data points.

The PhyClone generative model without outliers is as follows.

$$\begin{aligned}
\mathbf{b}|\alpha &\sim \text{CRP}(\alpha) \\
F = (E', V')|\mathbf{b} &\sim \text{Uniform}(\mathcal{F}_{|\boldsymbol{b}|}) \\
V|V' &= V \cup \{r\} \\
E|E' &= E' \cup \{(r, u) : \text{indegree}(u) = 0\} \\
T &= (E, V) \\
\boldsymbol{\kappa}|T &= \kappa \mathbf{1}_{|V|} \\
\boldsymbol{\rho}|\boldsymbol{\kappa} &\sim \text{Dirichlet}(\boldsymbol{\kappa})
\end{aligned}$$

where the notation $\mathbf{1}_k$ indicates the vector of ones with dimension k.

Thus, we sample a partition $\mathbf{b}$ from a CRP. Given $\mathbf{b}$ we sample a forest uniformly at random from the set of forests of size $|\mathbf{b}|$. We then deterministically connect all root nodes in the forest to a dummy node r to create a tree. Next we sample a vector, $\boldsymbol{\rho}$, of clonal prevalences from a Dirichlet distribution of dimension $|\mathbf{b}| = |V|$. Each node is associated with a clonal prevalence in this vector. This model can be trivially generalized to multi-region sequencing by sampling the clonal prevalence of each region from a Dirichlet distribution. For simplicity we will assume a single sample in this section to keep the notation uncluttered. We also note that $\boldsymbol{\rho}$ depends on T since the dimensionality of $\boldsymbol{\kappa}$ is determined by $|V|$. To simplify notation we do not write this dependence explicitly in some equations, but it should always be understood.

Next we define how to compute the cellular prevalence of a mutation, which will be used in the PyClone emission density for the allele counts of a mutation. Let

- T_v denote the sub-tree of T rooted at node v.
- V_v denote the set of nodes in T_v.
- C_v denote the set of children nodes of v.
- $v_n \in V$ denote the node associated with block $b \in \mathbf{b}$ such that $n \in b$, that is mutation n is in cluster b.
- $\bar{\rho}_v = \sum_{v' \in V_v} \rho_{v'} = \rho_v + \sum_{v' \in C_v} \bar{\rho}_{v'}$

In this notation $\bar{\rho}_{v_n}$ represents the cellular prevalence of mutation n. With this notation the data likelihood is

$$p(X|\boldsymbol{\rho}, \mathbf{b}, T) = \prod_{n=1}^{N} f(x_n|\bar{\rho}_{v_n}),$$

where $f(x|\bar{\rho})$ is the PyClone emission density defined in supplementary section S1.1.

Thus, the joint density is given by

$$\begin{aligned} p(X, \mathbf{b}, T, \boldsymbol{\rho}) &= p(\mathbf{b}|\alpha)p(T|\mathbf{b})p(\boldsymbol{\rho}|\boldsymbol{\kappa})p(X|\boldsymbol{\rho}, \mathbf{b}, T) \\ p(\mathbf{b}|\alpha) &\propto \alpha^{|\mathbf{b}|} \prod_{b \in \mathbf{b}} (|b| - 1)! \\ p(F|\mathbf{b}) &= \frac{1}{(|\mathbf{b}| + 1)^{|\mathbf{b}| - 1}}. \end{aligned}$$

The normalisation of $p(F|\mathbf{b})$ can be derived from Cayley's formula for the number of rooted trees. We use the observation that any rooted forest over K nodes can be turned into a rooted tree over $K + 1$ nodes by setting all root nodes to be children of a new dummy root node.

S1.3.1 Penalizing multi-rooted final tree topologies

One adjustment to the above is made when computing the prior density of the tree that follows after the addition of all datapoints has been completed during sampling. This adjustment was found to be necessary to address a bias towards trees with multiple children beneath the dummy node, referred to as sub-roots, which once removed would represent a multi-rooted tree. The adjustment itself is to penalize the likelihood of trees with more than one sub-root. Let the root adjustment term be $p(r|\mathbf{b})$, where the constant C is chosen such that the single rooted tree is 1000 times more likely than the two root tree etc., and where r is the number of sub-roots:

$$\begin{aligned} C &= 1000 \\ Z &= \sum_{i=1}^{|\boldsymbol{b}|} 1^{|\boldsymbol{b}|} \frac{1}{C^{i-1}} \\ p(r|\mathbf{b}) &= \frac{1}{ZC^{r-1}}. \end{aligned}$$

Let w represent the number of ways that the current topology with its number of nodes, sub-roots, and sub-root topologies can exist; which is computed by applying Cayley's formula for the number of rooted trees on each of

these sub-root trees and computing the product of the results. Let the set of sub-root trees be $\boldsymbol{S}$. The normalisation of $p(F|\mathbf{b})$ with the root adjustment term then becomes:

$$p(w|\mathbf{b}) = \prod_{s_r \in \boldsymbol{S}} (|s_r| + 1)^{|s_r|}$$
$$p(F|\mathbf{b}) = \frac{1}{w} p(r|\mathbf{b}).$$

S1.4 Collapsed distribution

For inference it is beneficial to work with the collapsed joint distribution, where we marginalise the node parameters $\boldsymbol{\rho}$. Let Δ_k denote the k simplex, $\Delta_k = \left\{\boldsymbol{\rho} \in \mathbb{R}_+^k : \sum \rho_i = 1\right\}$.

$$\begin{aligned} p(X, \mathbf{b}, T) &= \int_{\Delta_{|V|}} p(X, \mathbf{b}, T, \boldsymbol{\rho}) d\boldsymbol{\rho} \\ &= p(\mathbf{b}|\alpha) p(T|\mathbf{b}) \int_{\Delta_{|V|}} p(\boldsymbol{\rho}|\boldsymbol{\kappa}) p(X|\boldsymbol{\rho}, \mathbf{b}, T) d\boldsymbol{\rho} \\ &= p(\mathbf{b}|\alpha) p(T|\mathbf{b}) \int_{\Delta_{|V|}} p(\boldsymbol{\rho}|\boldsymbol{\kappa}) \prod_{n=1}^{N} f(x_n|\bar{\rho}_{v_n}) d\boldsymbol{\rho} \end{aligned}$$

Computing $\int p(\boldsymbol{\rho}|\boldsymbol{\kappa}) \prod_{n=1}^{N} f(x_n|\bar{\rho}_{v_n}) d\boldsymbol{\rho}$ is non-trivial because of the dependence on the tree structure.

We note that $\boldsymbol{\rho}$ has a Dirichlet distribution thus $p(\boldsymbol{\rho}|\boldsymbol{\kappa}) = c(\boldsymbol{\kappa}) \prod_{v \in V} \rho_v^{\kappa_v - 1}$. Thus, we can reorganise the computation. In the following we write $n \in v$ to denote that mutation n is in the block $b \in \mathbf{b}$ associated with node v.

$$\begin{aligned} \int_{\Delta_{|V|}} p(\boldsymbol{\rho}|\boldsymbol{\kappa}) \prod_{n=1}^{N} f(x_n|\bar{\rho}_{v_n}) d\boldsymbol{\rho} &= \int_{\Delta_{|V|}} p(\boldsymbol{\rho}|T) \times \prod_{v \in V} \prod_{n \in v} f(x_n|\bar{\rho}_v) d\boldsymbol{\rho} \\ &\propto \int_{\Delta_{|V|}} \left[\prod_{v \in V} \rho_v^{\kappa_v - 1} \prod_{n \in v} f(x_n|\bar{\rho}_v) \right] d\boldsymbol{\rho} \\ &= \int_{\mathcal{S}_T} \left[\prod_{v \in V} \left(\bar{\rho}_v - \sum_{v' \in C_v} \bar{\rho}_{v'} \right)^{\kappa_v - 1} \prod_{n \in v} f(x_n|\bar{\rho}_v) \right] d\bar{\boldsymbol{\rho}} \end{aligned}$$

We perform a change of variable into the $\bar{\rho}_v$ parameterization and consequently also change the domain of integration into $\mathcal{S}_T = \left\{ \bar{\boldsymbol{\rho}} \in \mathbb{R}_+^{|V|} : \sum_{v' \in C_v} \bar{\rho}_{v'} \leq \bar{\rho}_v \leq 1 \right\}$.

To make further progress we use a discrete approximation for $\bar{\boldsymbol{\rho}} = (\bar{\rho}_1, \ldots, \bar{\rho}_{|V|})$. Specifically we assume that for a grid of size $L+1$, $\bar{\rho}_v \in \Phi = \left[0, \frac{1}{L}, \ldots, \frac{L-1}{L}, 1\right]$. We write $\mathcal{S}_{T,L}$ for the corresponding discretization of $\mathcal{S}_T$.

S1.5 Algorithm

Please note, while the algorithm is described recursively below, the implementation itself is achieved via dynamic programming so as to capture further efficiency.

Let $R_v(\bar{\rho})$ denote the likelihood of a sub-tree rooted at $v \in V$ when the mutations located at the root of this sub-tree have cellular prevalence $\bar{\rho} \in \Phi$. Let $\ell_v(\bar{\rho}_v) = \prod_{n \in v} f(x_n|\bar{\rho}_v)$. If we denote the sub-tree of T rooted at v by $T_v = (V_v, E_v)$, this has the form:

$$\begin{aligned} R_v(\bar{\rho}) &= \sum_{\bar{\boldsymbol{\rho}} \in \mathcal{S}_{T,L} : \bar{\rho}_v = \bar{\rho}} \prod_{v' \in V_v} {\rho_{v'}}^{\kappa - 1} \ell_{v'}(\bar{\rho}_{v'}) \\ &= \sum_{\bar{\boldsymbol{\rho}} \in \mathcal{S}_{T,L} : \bar{\rho}_v = \bar{\rho}} \prod_{v' \in V_v} \left(\bar{\rho}_{v'} - \sum_{v'' \in C_v} \bar{\rho}_{v''} \right)^{\kappa - 1} \ell_{v'}(\bar{\rho}_{v'}). \end{aligned}$$

We need to show that $R_v(\cdot)$ can be efficiently computed from the recursions $\{R_{v'}(\cdot) : v' \in C_v\}$. This is done in three steps. In this section, we show the steps taken by the algorithm, and we analyse and justify them in supplementary section S1.5.1. The pseudo-code for the marginalisation algorithm consists, for all $v \in V$ in post-order with respect to T:

1. A first sub-recursion, which iteratively incorporates the sub-recursions obtained for each of the $|C_v|$ children of node v:

$$D_v^1(\bar{\rho}) = R_{v_1}(\bar{\rho}),$$
$$D_v^k(\bar{\rho}) = \sum_{\bar{\rho}' \in \Phi : \bar{\rho}' \leq \bar{\rho}} R_{v_n}(\bar{\rho}) D_v^{k-1}(\bar{\rho} - \bar{\rho}'),\ k \in (2, \ldots, |C_v|).$$

2. A second sub-recursion to marginalise the sum of the children's total prevalence, $\sum_k \bar{\rho}_{v_k} = \bar{\rho}'$, while taking into consideration the prior contribution based on the residual prevalence assigned to the current node v:

$$S_v(\bar{\rho}) = \sum_{\bar{\rho}' \in \Phi : \bar{\rho}' \leq \bar{\rho}} (\bar{\rho} - \bar{\rho}')^{\kappa - 1} D_v^{|C_v|}(\bar{\rho}'),$$

which, when $\kappa = 1$, reduces to:

$$S_v(0) = 0,$$
$$S_v(\bar{\rho}) = D_v^{|C_v|}(\bar{\rho}) + S_v\left(\bar{\rho} - \frac{1}{L}\right),\ \bar{\rho} \in \left(\frac{1}{L}, \ldots, \frac{L}{L}\right).$$

3. Finally:

$$R_v(\bar{\rho}) = \ell_v(\bar{\rho}) S_v(\bar{\rho}).$$

We obtain the likelihood of interest (marginalising the prevalence and given a tree) by enforcing the constraint that the sum of all clonal prevalence is one:

$$\int_{\Delta_{|V|}} p(\boldsymbol{\rho}|\boldsymbol{\kappa}) \prod_{n=1}^{N} f(x_n|\bar{\rho}_{v_n}) d\boldsymbol{\rho} = R_r(1),$$

where $v = r$ is the root of the tree.
Pseudocode for the algorithm as a dynamic programming implementation is provided in Algorithm 1. The asymptotic runtime of this algorithm is as follows:

$$\mathcal{O}(V(NL + CSL^2)).$$

Where V is the number of nodes in the tree, N is the number of mutations, L is the grid size, C is the maximum out-degree of tree nodes, and S is the number of samples.

S1.5.1 Justification

The algorithm is based on the following decomposition:

$$R_v(\bar{\rho}) = \ell_v(\bar{\rho}) \times S_v(\bar{\rho})$$
$$S_v(\bar{\rho}) = \sum_{\bar{\rho}' \in \Phi : \bar{\rho}' \leq \bar{\rho}} (\bar{\rho} - \bar{\rho}')^{\kappa - 1} \times D_v^{|C_v|}(\bar{\rho}')$$
$$D_v^{|C_v|}(\bar{\rho}') = \sum_{\{\bar{\rho}_{v_1}, \ldots, \bar{\rho}_{v_{|C_v|}} : \sum_k \bar{\rho}_{v_k} = \bar{\rho}'\}} \prod_{k=1}^{|C_v|} R_{v_k}(\bar{\rho}_{v_k}).$$

In this decomposition, $\bar{\rho}'$ can be interpreted as the left hand side of the constraint imposed by the domain of integration $\mathcal{C}_T$ over the prevalences of the children:

$$\underbrace{\sum_{k=1}^{|C_v|} \bar{\rho}_{v_k}}_{\bar{\rho}'} \leq \underbrace{\bar{\rho}_v}_{\bar{\rho}}.$$

S1.6 Inference

To simultaneously infer the posterior distribution of T and $\mathbf{b}$ we use a sequential Monte Carlo (SMC) algorithm. The SMC algorithm works by evolving a set of particles which are iteratively extended and reweighed, such that the final iteration yields an approximation of the posterior. We assume that a permutation, σ, of $[N]$ has been given. At algorithmic time t of the SMC procedure we add data point $X_{\sigma(t)} = x_t$. We will grow the rooted forest from the bottom up. Because we use a fixed order of the data points, not all trees will be accessible using this procedure. Specifically, if $x = \sigma_i$ and $y = \sigma_j$ where $i < j$ then any tree where x is an ancestor of y cannot be reached. In the next sections we describe the basic SMC procedure, and later describe how to fix the ordering problem using a Particle Gibbs (PG) sampler.

S1.6.1 Target density

To specify our SMC algorithm, we need to define as sequence of target densities, γ_t. Importantly, the final target densities γ_N must be proportional to the distribution we wish to sample from i.e. the posterior distribution. Let T_t be the rooted tree generate at algorithmic time t by connecting all existing root nodes in the forest to a dummy root. We let our target density be

$$\begin{aligned}\gamma_t(x_{1:t}) &= p(X_t, \mathbf{b}_t, T_t) \\ &= p(\mathbf{b}_t)p(T_t|\mathbf{b}_t)p(X_t|T_t).\end{aligned}$$

If $|\mathbf{b}_t| = |\mathbf{b}_{t-1}|$, we allocate a data point to an existing cluster b and

$$\frac{\gamma_t(x_{1:t})}{\gamma_{t-1}(x_{1:t-1})} = |b| \frac{p(X_t|T_t)}{p(X_{t-1}|T_{t-1})},$$

otherwise we create a new cluster and

$$\frac{\gamma_t(x_{1:t})}{\gamma_{t-1}(x_{1:t-1})} = \alpha \times \frac{(|\mathbf{b}_{t-1}|+1)^{|\mathbf{b}_{t-1}|-1}}{(|\mathbf{b}_{t-1}|+2)^{|\mathbf{b}_{t-1}|}} \times \frac{p(X_t|T_t)}{p(X_{t-1}|T_{t-1})}$$

It can be checked that

$$\begin{aligned}\gamma_N(x_{1:N}) &= \gamma(x_1) \prod_{t=2}^{N} \frac{\gamma_t(x_{1:t})}{\gamma_{t-1}(x_{1:t-1})} \\ &= \frac{\alpha^{|\mathbf{b}|} \prod_{b \in \mathbf{b}} (|b|-1)!}{(|\mathbf{b}|+1)^{|\mathbf{b}|-1}} p(X|T) \\ &= p(X, \mathbf{b}, T).\end{aligned}$$

satisfying the criteria that our final target density is proportional to the posterior distribution.

S1.6.2 Proposal function

As part of the SMC algorithm we also need to define a proposal kernel, which extends the state at time $t-1$ to a new state at time t. To propose a new state at time t we consider all possible states which can be reached by

1. Adding data point to an existing node.
2. Creating a new node choosing a possibly empty subset of children from the existing root nodes.

There are several ways in which to perform these action. The simplest approach is to randomly choose to perform steps 1 or 2, and then randomly sample a node if we choose 1 or randomly select a set of children if we choose 2. We refer to this as the bootstrap kernel in the code. While computationally inexpensive, this approach will tend to propose very poor trees and require many particles.

Alternatively we could enumerate all possible choices and compute the probability of the trees which result. We can then sample proportional to these probabilities. We refer to this as the fully-adapted kernel in the code. This approach becomes computationally expensive as we need to enumerate all possible subsets of children if we start a new node.

In practice we use a hybrid approach. We randomly choose whether to join an existing node or start a new node. When adding a mutation to an existing node we compute the probability of the resulting tree with the mutation attached at each node. When adding a mutation to a new node we randomly sample the set of children. We refer to this as the semi-adapted kernel in the code. This approach provides a compromise between the quality of trees proposed and the computational complexity.

S1.6.3 Particle Gibbs sampler

We treat the ordering of data points added at algorithmic time t as fixed before running the SMC procedure. This limits the utility of the SMC algorithm, as only a subset of trees can be reached. For example, the first element of σ can never be a root node unless the forest has only a single node. To address this issue we use a particle Gibbs (PG) sampler to embed our SMC procedure in a more general Markov chain Monte Carlo sampler. With a slight abuse of notation we will use $p(X, T)$ to denote $p(X, \boldsymbol{b}, T)$ where it understood each node in the tree is associated with a cluster of mutations. We define $\Sigma(T)$ to be the set of permutations which could generate T using the SMC inference scheme. Instead of constructing an SMC algorithm to target the posterior $p(T|X)$ directly, we alternate between using conditional SMC to target $p(T|X, \sigma)$ and sampling from $p(\sigma|T)$:

$$
\begin{aligned}
p(T|X,\sigma) &\propto p(T, X, \sigma) \\
&= p(\sigma|T)p(X|T)p(T) \\
p(\sigma|T) &= \frac{1[\sigma \in \Sigma(T)]}{\sum_{\sigma} 1[\sigma \in \Sigma(T)]} \\
&= \frac{1[\sigma \in \Sigma(T)]}{Z(\sigma)},
\end{aligned}
$$

where we define $1[\sigma \in \Sigma(T)]$ to be one if and only if σ can be generated by the following recursive algorithm.

1. For a leaf node, return a randomly shuffled list of its assigned mutations.
2. For an internal node, recursively collect the sigma lists returned from processing its child nodes. Interleave these child-node lists using a bridge shuffle to create a flat sigma list; then collect the datapoints assigned to the current node, shuffle their order, and append them to the end of the current sigma list.
3. At the root node, proceed as above in the internal node case, with the added step of also collecting the outlier datapoints (should any exist), shuffling their order and interleaving the outliers with the sigma list.

S1.6.4 Particle Gibbs sub-tree sampling

The above procedure can be easily modified to update sub-trees instead of the whole tree. This can alleviate the well known degeneracy problem for PG samplers. Degeneracy refers to the problem that for long sequences all particles will eventually trace their genealogy back to the conditional path in the swarm. This limits the ability of the sampler to change states that appear early in the SMC iterations. In our case this means that deeper nodes in the tree will tend to change less frequently. By re-sampling sub-trees we can shorten the SMC paths and more efficiently update deeper nodes in the tree.

To select a sub-tree for updating, we randomly sample a mutation and find the node the mutation is assigned to. We then take the parent of that node as the root of the sub-tree which we will update. Next we use the PG sampler described above to update sub-tree. At the final iteration we reinsert the sub-tree into the original tree and compute the full tree likelihood to use in the computation of the importance weights. This ensures the final target density in the conditional SMC is proportional to the target posterior distribution.

One non-obvious aspect of the above algorithm is the mechanism for choosing the node to use as the sub-tree root. We choose a mutation at random so that the update does not depend on the current state of the Monte Carlo sampler. We choose to use the parent of the associated node as the sub-tree root in order to allow the sampler to update the entire forest. If we did not do this, we could never change the attachments to the dummy root.

S1.7 Outliers

A major constraint of the model described thus far is that mutations are assumed to be propagated to all nodes that descend from the node of origin. This constraint can easily be violated if copy number alterations remove the mutation in a descendant. Furthermore, we may have mutations which may be noisy either due to erroneous copy number or incorrect allele counts due to alignment errors. To address these issues the above model can be extended with an outlier state. Each mutation is assigned a prior probability of being an outlier, ν_n. We define a binary variable o_n which indicates whether mutation n is an outlier. If a mutation is not an outlier it joins a node in the tree and contributes to the likelihood as above. If the mutation is an outlier it has the standard PyClone

likelihood where we assume it has a cellular prevalence drawn from a Uniform distribution. We marginalise the cellular prevalence in this case using numerical integration. The updated joint likelihood becomes

$$\begin{aligned} p(X, \mathbf{b}, T, \mathbf{o}) = & \prod_{n=1}^{N} [\nu_n \int f(x_n|\rho) d\rho]^{\mathbb{I}(o_n=1)} \\ & \times \prod_{n=1}^{N} p(\mathbf{b}|\alpha) p(T|\mathbf{b}) \int p(\boldsymbol{\rho}|\boldsymbol{\kappa}) \\ & \times \prod_{n=1}^{N} [(1-\nu_n) f(x_n|\bar{\rho}_{v_n})]^{\mathbb{I}(o_n=0)} d\boldsymbol{\rho} \end{aligned}$$

The modifications to the inference procedure are straightforward. When proposing a new state we now include the possibility that a mutation is an outlier. When re-sampling σ we randomly permute the set of outliers and interleave them with the permuted values from the tree using a bridge shuffle. The algorithm for counting the number of permutations can be easily modified to account for this. Finally, when performing sub-tree updates we include all outliers in the set of mutations to be considered for the sub-tree update.

S1.7.1 Outlier prior probability assignment from clustered data

PhyClone can assign prior outlier probabilities for clusters given a clustered input file which includes: SNV genomic position (chromosome and position), and SNV or cluster level cellular prevalence information. Given the appropriate input, PhyClone will, in a manner not dissimilar from that employed by CONIPHER [1], assign clusters with either a high or low outlier prior probability (both levels are user-defined, default to 0.0001 for low and 0.4 for high outlier prior probability).

The first stage in the data informed approach is to first identify the cluster most likely to be truncal in the phylogeny, this is achieved by selecting the cluster with the highest cellular prevalence across samples. Should there be multiple candidate clusters from this primary filtering, a second stage is employed where the mean of all cellular prevalence across all samples are computed for each candidate cluster; the truncal cluster will then be the cluster with the maximum mean cellular prevalence from the truncal candidates.

Following truncal cluster selection, a background distribution of mutations per unique chromosome is established from the truncal cluster. For each non-truncal cluster, given its set of mutations m, we compute the expected number of unique mutations on a chromosome by making 10,000 random draws of size $|m|$ from the truncal background distribution. From these 10,000 random trials, the number of trials with fewer unique chromosomes than what is observed (N_u) will be used to compute the resulting p-value ($\frac{N_u}{10000}$). Clusters with a computed p-value < 0.01 will be defined as more likely to be lost due to a closer than expected genomic locality.

S2 Implementation Details

PhyClone is implemented in Python 3, and has been developed for and tested exclusively in the Linux operating system. Given the computational complexity of the Particle Gibbs sequential Monte Carlo (PG-SMC) framework, a variety of pre-computations and caching strategies are employed in the implementation of PhyClone. Furthermore, to ensure that the PhyClone mathematical model is accurately translated into code, an extensive suite of both unit and integration tests are implemented alongside the PhyClone code-base.

S2.1 Pre-computations

As a feature of the PhyClone model's discretization of CCF values, we are able to pre-compute the PyClone emission density for all mutations (or clusters of mutations) and samples. We are able to further extend these pre-computations into the realm of the outlier marginal probabilities, which are themselves pre-computed given the dimensions of the discretization grid, the outlier prior probabilities, and the aforementioned pre-computed PyClone emission density values. These values are then cached and held within the DataPoint class, which are retained over the entirety of a PhyClone analysis run, which allows us to only have to perform these computationally expensive procedures once.

S2.2 Caching

Though the α concentration hyper-parameter value is resampled between PhyClone iterations, we note that within each iteration the value is held steady and thus we are able to employ various caching strategies. For ease and clarity, in the following, the pre-computed PyClone emission density values for a mutation/cluster of mutations will be referred to as a DataPoint (in reference to the class that holds the values). All caches described below make use of the Least Recently Used (LRU) cache ejection strategy as this allows for an upper limit on cached values, thus protecting memory resources.

S2.2.1 Convolution result caching

The bottom-up construction procedure of the PhyClone SMC algorithm design allows for the interpretation of the marginalisation algorithm as a series of convolutions over post-order subtrees. Then, given that the number of DataPoints is finite, and each DataPoint can only ever be assigned to a single tree node at a time, it becomes clear to see that while the number of combinations of these convolutions is large - they are still in fact, finite.

This led to the realisation that a dramatic decrease in runtime could be found by intelligently caching the results of these convolutions. Importantly, with convolution being a commutative operation, the order in which a node's children are convolved does not matter, which further restricts the number of unique convolutions possible - as long as the sub-tree order of convolutions is respected.

Thus, when caching the result of a convolution, it worked best to create a hash that could represent the unique nesting of sets of values that led to the result. Two levels of convolution were thus cached:

- Two node convolution: i.e. the result of two nodes being convolved together (akin to incorporating two child nodes together).
- The result of a sub-tree root's nested convolution.

The form of the hash presented a further challenge, as the best descriptive form for the convolution results were the values that were initially convolved. At a high-level, to solve this problem two array hashing classes were developed: a two array hashing class and a list of arrays hashing class; both of which would internally rely on some functionality to hash an individual array of values.

Thus, the challenge became having to compute a hash for an array of values in a way that would ensure the following: collision safety, minimal impact on memory, and also still represent a speedup over the $O(n^2)$ cost of direct convolution.

This was achieved by taking the view that the array of values could be used as exactly what it is at a low-level; a byte-array. A caveat being that all arrays must be contiguous in memory to ensure equivalent hashes are created for equivalent arrays of values. With a byte-array one could then use any number of extant and expedient non-cryptographic hashing algorithms to produce a unique hash string; PhyClone makes use of the xxHash3 algorithm `https://github.com/Cyan4973/xxHash`.

S2.2.2 Tree caching

Note: The following will refer to the PhyClone Tree class, with the capitalization in the word "Tree" being operative in its denotation.

The next level of result caching came about from the observation that within a PhyClone iteration, the data-order σ is set, thus while the space of possible tree topologies is large - it is still restricted and finite. Furthermore, due to the random-sampling nature of the PG-SMC, there was bound to be some overlap in tree topologies drawn. Meanwhile, the construction of a PhyClone Tree requires the combination of various levels of computationally expensive routines. Thus motivating the need to cache PhyClone Trees.

Given that a full PhyClone Tree (as implemented in a Python class) can have a non-insignificant impact on space complexity, the TreeHolder class was developed. The TreeHolder class retains all the information required to re-establish the full PhyClone Tree class, as well as all the necessary information required to be used as a low-memory stand-in representation of a PhyClone Tree. Importantly for further downstream caching strategies, the TreeHolder class includes sufficient information to compute likelihoods of both partial and complete trees (the distinction being that a partial tree does not yet have all DataPoints included).

These features allow the TreeHolder class to be cached with minimal impact on space complexity, and thus drastically reduces the number of redundant trees constructed over the duration of a PhyClone run.

S2.2.3 Proposal distribution caching

During the conditional path construction step of the SMC algorithm, DataPoints are added iteratively to the tree, establishing a path of particles based on these partial trees along the way.

These particles are later drawn from and re-sampled over the course of the sampling step of the PG-SMC. While this sampling occurs, the likelihood of a tree is established through the proposal distribution; the computation of which can be incredibly expensive as its complexity grows by the number of root-nodes present in the current tree.

However, many of these proposal distributions will have the exact same set of trees involved in their initialization: for the semi-adaptive sampler, this is the set of all trees possible from adding a DataPoint to an extant node; while for the fully-adaptive sampler, this is simply the set of all possible trees derived from incorporating the DataPoint. Thus, a significant performance increase could be seen by managing to cache these distributions.

The caching of the proposal distributions relies heavily on the fact that the TreeHolder class offers a low-memory version of the PhyClone Tree, while still offering all of the required information to compute likelihoods. With this being the case, the proposal distributions can be simply cached and drawn upon when seeking the likelihood of a Tree with a DataPoint that matches the one used to originally compute the proposal distribution.

S2.3 Code Correctness

To ensure that the PhyClone codebase does not begin introducing and propagating hard-to-catch errors an extensive suite of tests was developed. Most relevant to the code's ability to correctly implement the PhyClone model are the exact enumeration and marginalisation likelihood comparative testing suites.

S2.3.1 Exact enumeration test

The exact enumeration testing suite involves a series of tests wherein small and tractable versions of the phylogenetic deconvolution problem have their posteriors exactly enumerated. The PhyClone sampling procedure is then allowed to run against the same set of simulated data-points, with the end inferred posteriors being compared against that of the exactly enumerated truth.

In order for an exact enumeration test to pass, all of PhyClone's inferred posterior values must be close to truth-values within a defined level of tolerance. The full exact enumeration testing suite is run against all available versions of PhyClone's proposal distribution kernels (semi-adapted, fully-adapted, and bootstrap).

While a unit test in name, the exact enumeration test allows us to evaluate if the complete PG-SMC sampler exhibits any inference breaking errors; though these errors themselves may have been propagated through various sources, such as the Tree class, the underlying distribution classes, or any other class or functionality that the PhyClone PG-SMC scheme relies on. As such, the exact enumeration test in fact behaves and demonstrates usefulness as an integration testing suite for many of PhyClone's most salient features.

S2.3.2 Marginalisation likelihood comparative test

PhyClone uses a marginalisation algorithm which allows for the estimation of clonal prevalence over a discretized grid of values, thus integrating out clonal proportion during tree likelihood scoring. To ensure its correctness, the marginalisation likelihood comparative test was developed.

The marginalisation likelihood comparative test is relatively simple in design. Given a static (ground-truth) tree topology and mutational clustering, compute the marginalised PhyClone likelihood score and compare it against the likelihood scored by some other estimator that samples over the clonal proportions.

The testing suite used in PhyClone for this purpose implements an importance sampler (IS). The IS works by making random draws from a Dirichlet distribution for possible clonal prevalence measures, and scores the likelihood of the proposed values given the tree, weighing the proposal by the likelihood ratio; and finally reporting the weighted-average likelihood taken over all iterations as its computed likelihood value. As the IS is an inherently inefficient sampler, during testing, the IS method is run for 1,000,000 iterations per trial.

Each individual marginalisation comparative test is run over a minimum of ten independently simulated trees (or trials), with each test varying the number of mutations, samples, or other pertinent tree feature. In order to pass a marginalisation comparative test, the IS likelihoods and PhyClone marginalised likelihoods must be found to have statistically equal distributions. In practice, the likelihood distribution equality is scored through an independent two-sample, two-tailed, T-test.

S3 Supplementary Figures

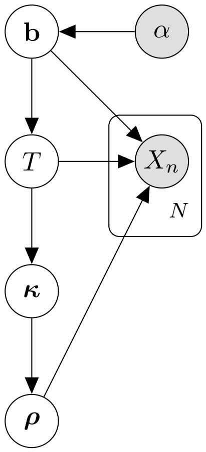

$$
\begin{aligned}
\mathbf{b}|\alpha &\sim \text{CRP}(\alpha)\\
T=(E,V)|\mathbf{b} &\sim \text{Uniform}(\mathcal{F}_{|\mathbf{b}|+1})\\
\boldsymbol{\kappa} &= \kappa\mathbf{1}_{|V|}\\
\boldsymbol{\rho}|\boldsymbol{\kappa} &\sim \text{Dirichlet}(\boldsymbol{\kappa})\\
\bar{\rho}_v|T,\boldsymbol{\rho} &= \sum_{v'\in V_v}\rho_{v'}\\
X_n|\mathbf{b},T,\boldsymbol{\rho} &\sim f(x_n|\bar{\rho}_{v_n})
\end{aligned}
$$

Figure S1: **Single Sample PhyClone Generative Model** Graphical model for the single sample PhyClone model. Parameter $\mathbf{b}$ denotes a partition of the data points $\{b \subset \{1,\ldots,N\} : \bigcup b = [N]\}$. We define $\mathcal{F}$ to be the space of all forests, and $\mathcal{F}_k = \{F=(E,V)\in\mathcal{F} : |V| = k\}$ to be the set of all trees with k nodes. $T=(E,V)$ denotes a rooted tree, that is, a graph with a directed edge set E and vertices set V. While $f(x_n|\bar{\rho}_{v_n})$ is the PyClone emission density as defined in supplementary section S1.1.

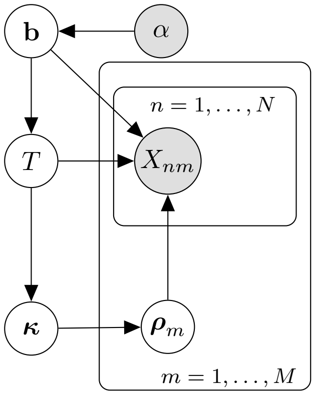

$$
\begin{aligned}
\mathbf{b}|\alpha &\sim \text{CRP}(\alpha)\\
T=(E,V)|\mathbf{b} &\sim \text{Uniform}(\mathcal{F}_{|\mathbf{b}|+1})\\
\boldsymbol{\kappa} &= \kappa\mathbf{1}_{|V|}\\
\boldsymbol{\rho}|\boldsymbol{\kappa} &\sim \text{Dirichlet}(\boldsymbol{\kappa}^M)\\
\bar{\rho}_{vm}|T,\boldsymbol{\rho}_m &= \sum_{v'\in V_v}\rho_{v'm}\\
X_{nm}|\mathbf{b},T,\boldsymbol{\rho}_m &\sim f(x_{nm}|\bar{\rho}_{v_n m})
\end{aligned}
$$

Figure S2: **Multiple Sample PhyClone Generative Model** Graphical model for the multi-sample PhyClone model. Parameter $\mathbf{b}$ denotes a partition of the data points $\{b \subset \{1,\ldots,N\} : \bigcup b = [N]\}$. We define $\mathcal{F}$ to be the space of all forests, and $\mathcal{F}_k = \{F=(E,V)\in\mathcal{F} : |V| = k\}$ to be the set of all trees with k nodes. $T=(E,V)$ denotes a rooted tree, that is, a graph with a directed edge set E and vertices set V. While $f(x_{nm}|\bar{\rho}_{v_n m})$ is the PyClone emission density as defined in supplementary section S1.1.

Algorithm 1 PhyClone Marginalisation Dynamic Programming Method

Input: $T = (V, E)$, $\bar{\boldsymbol{\rho}} = (\bar{\rho}_1, \ldots, \bar{\rho}_{|V|})$, X = observed copy number and read count data
Output: Node parameter marginalised likelihood of T

procedure Tree-Likelihood-Marginalization(T, $\bar{\boldsymbol{\rho}}$, X)
  for $v \in V$, post-order from T **do**
    $T_v \Leftarrow (V_v, E_v)$
    $R_v \Leftarrow$ Compute $R(T_v, \bar{\boldsymbol{\rho}}_v)$
  end for
end procedure

function Compute $R(T_v, \bar{\boldsymbol{\rho}}_v)$
  $\ell_v \Leftarrow$ Compute $\ell_v(\bar{\boldsymbol{\rho}}_v)$
  $C_v \Leftarrow$ child nodes of v
  if $|C_v| = 0$ **then**
    return ℓ_v
  else
    $S_v \Leftarrow$ Compute $S(C_v)$
    return $\ell_v \times S_v$
  end if
end function

function Compute $S(C_v)$
  $D \Leftarrow$ Compute $D(C_v)$
  $S[1] \Leftarrow D[1]$
  for $i \in (2, \ldots, |D|)$ **do**
    $S[i] \Leftarrow D[i-1] + D[i]$
  end for
  return S
end function

function Compute $D(C_v)$
  $D \Leftarrow R_{C_v[1]}$
  for $k \in (2, \ldots, |C_v|)$ **do**
    $D \Leftarrow D * R_{C_v[k]}$
  end for
  return D
end function

function Compute $\ell_v(\bar{\rho})$
  $\ell_v \Leftarrow 1$
  for $n \in v$ **do**
    $\ell_v \Leftarrow \ell_v \times$ PyClone$(x_n | \bar{\rho}_{v_n})$
  end for
  return ℓ_v
end function

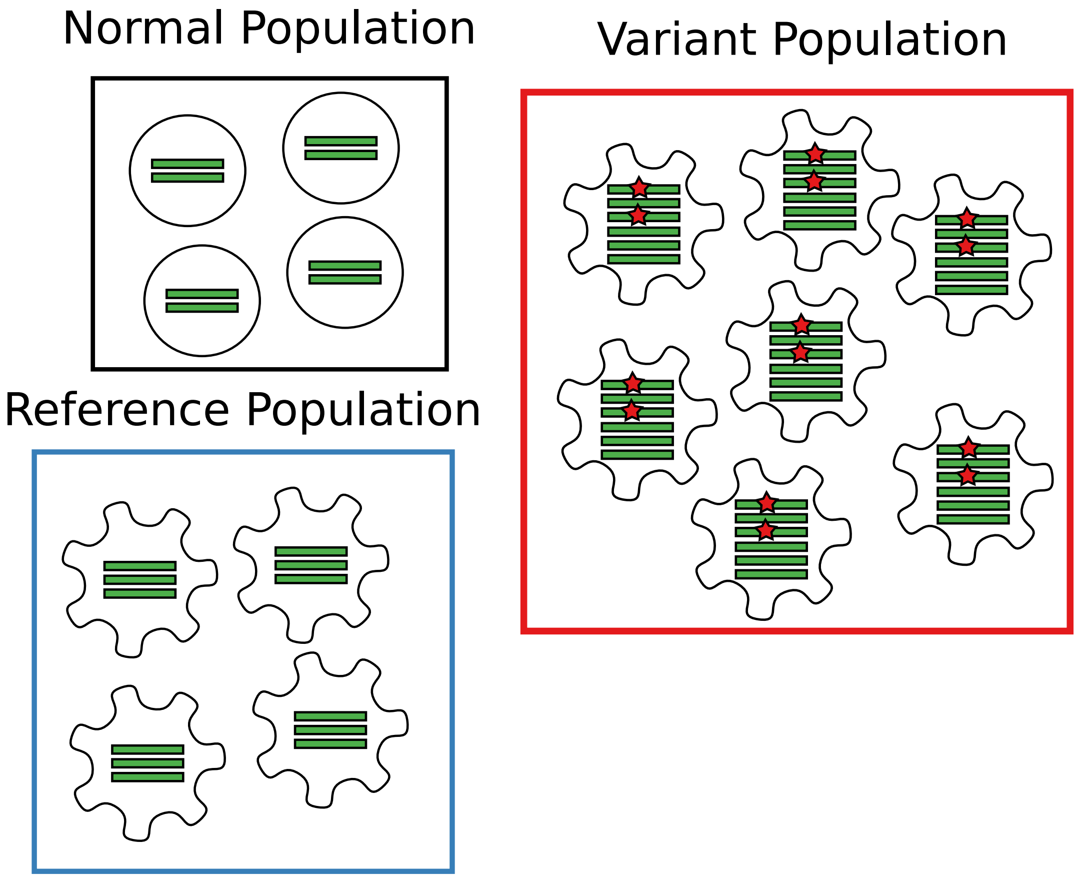

Figure S3: **Population structure assumed by the PyClone emission density**. Circular shaped cells are healthy cells, while irregular shaped cells are cancerous. The green bars are indicate chromatids and the red stars indicate a mutation. These are all assumed to overlap the loci which contains the mutation.

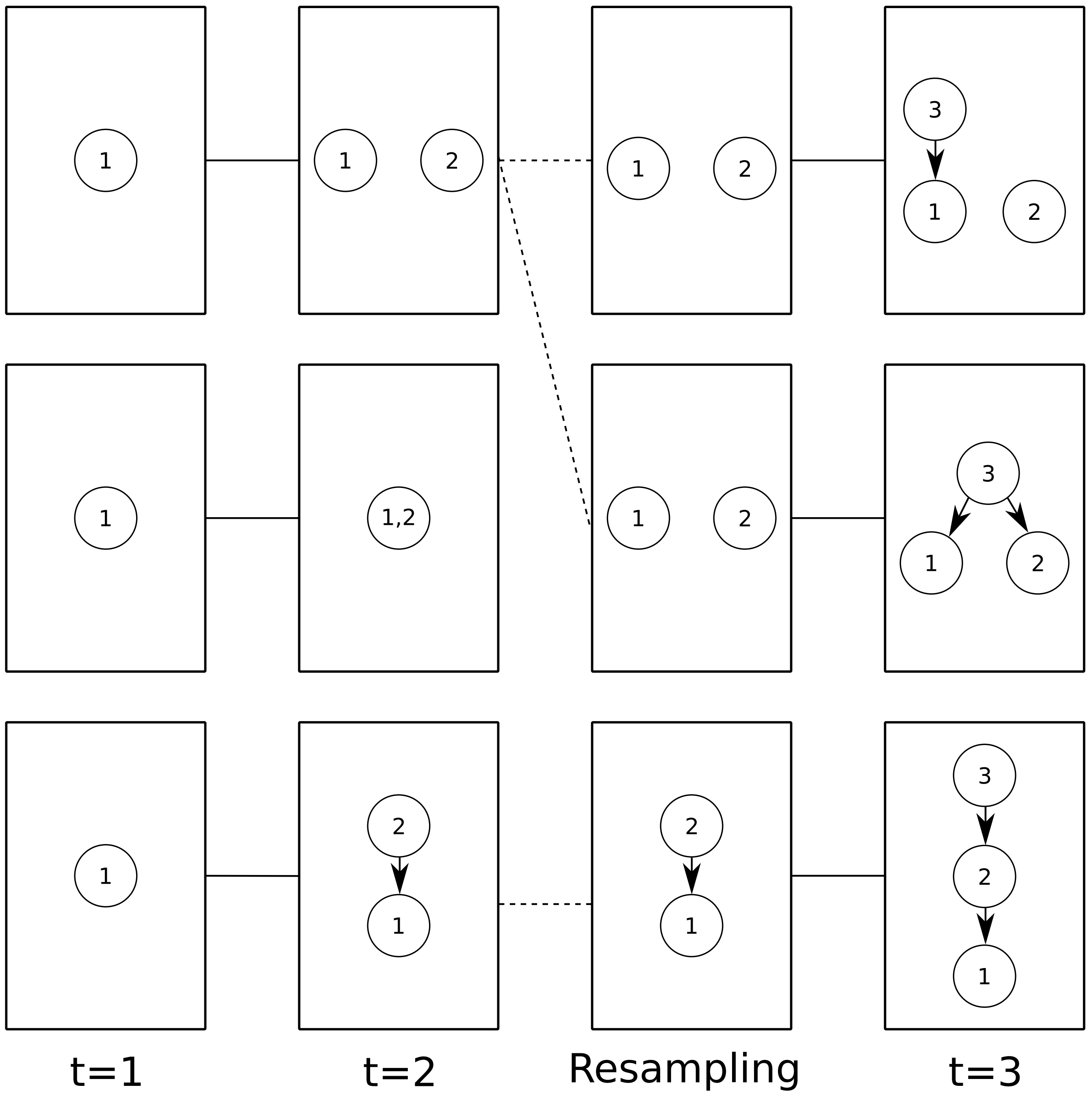

Figure S4: **Sequential Monte Carlo Sampling**. Diagram for the high-level view of the SMC sampling procedure.

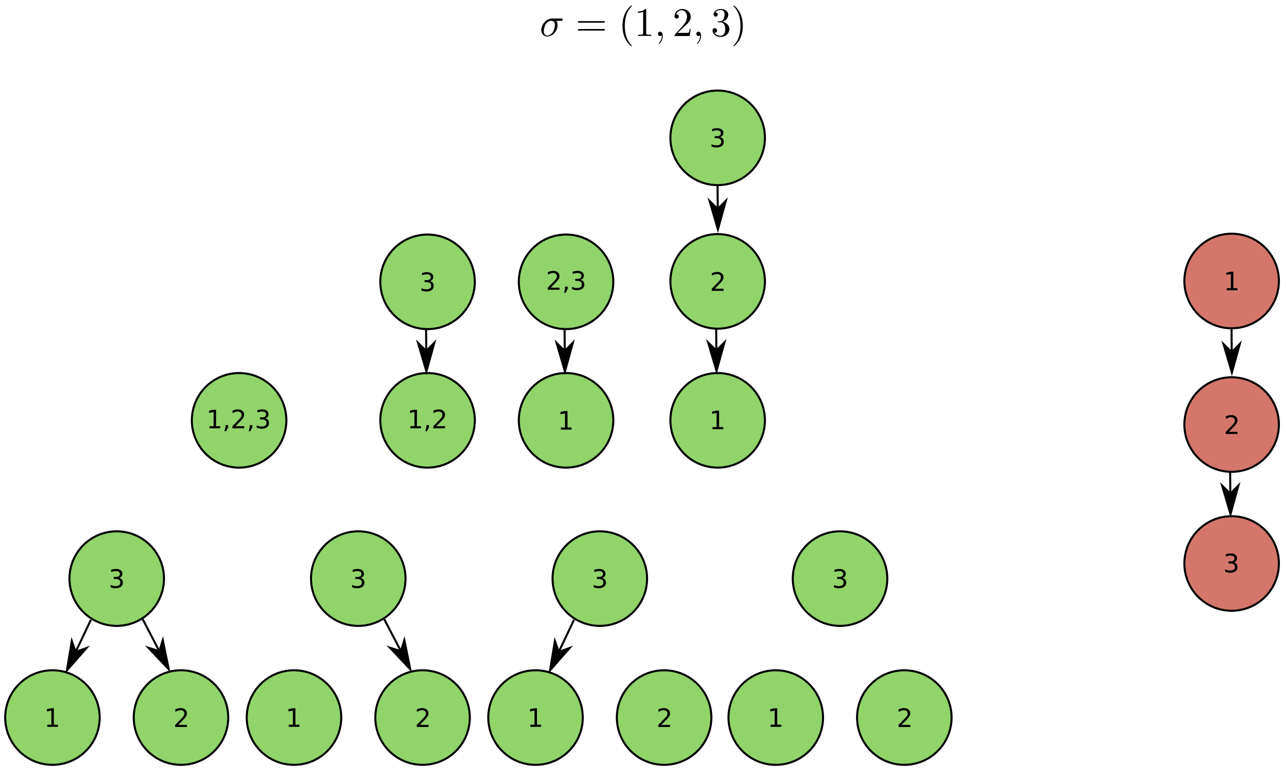

Figure S5: **SMC, Not all trees are reachable**. Graphical representation of how the data point ordering given to an SMC method can impact its end result when building up from the root. In this figure the tree on the right (in red), is unreachable by pure SMC due to the given data ordering; while all of the topologies on the left (in green), represent the topologies reachable by a pure SMC approach with the given data ordering.

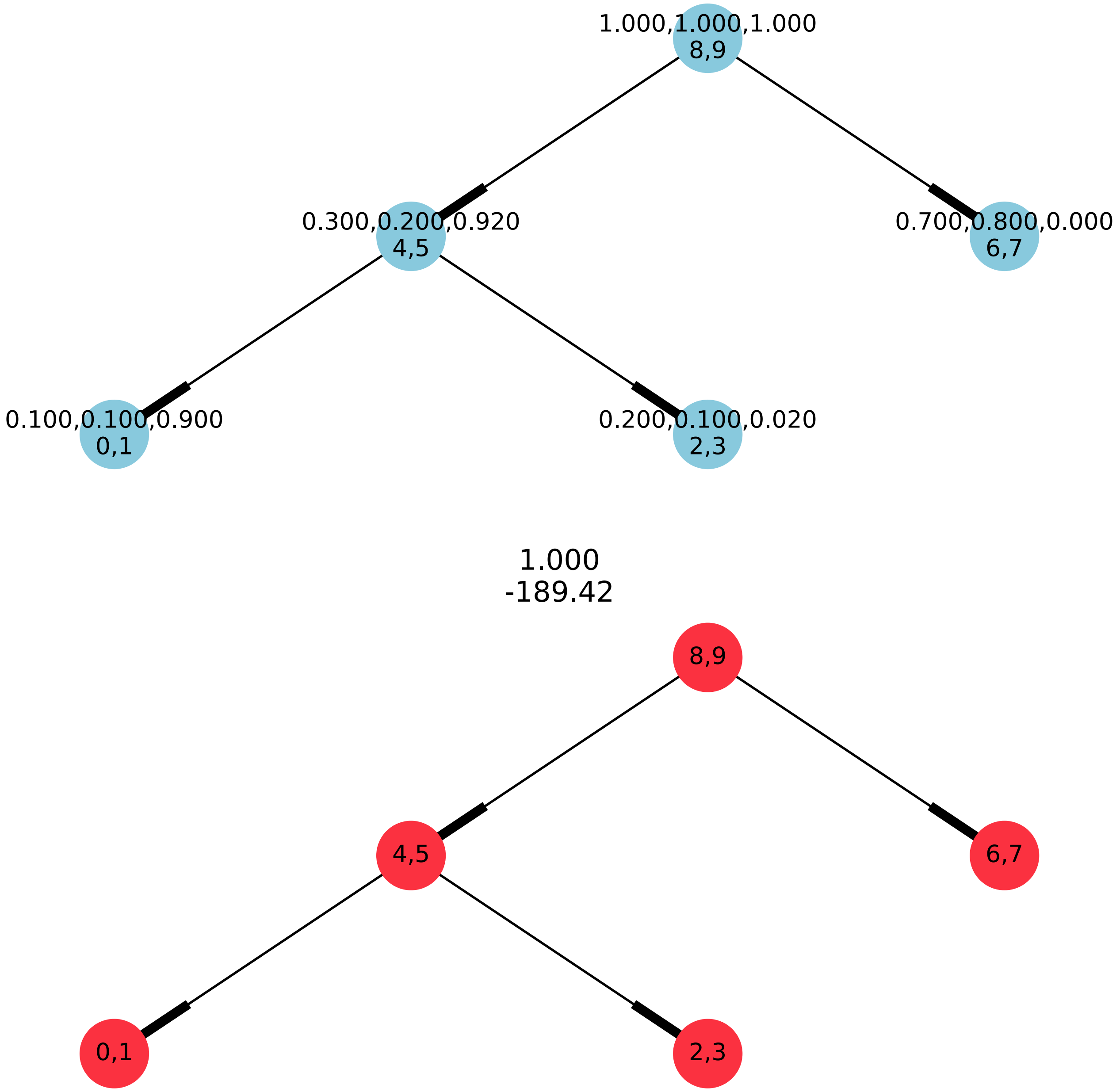

Figure S6: **SMC given a good data ordering**. Graphical representation of how the data point ordering given to an SMC method can impact its end result when building up from the root. Data ordering given as $\sigma = (0, 1, \ldots, 8, 9)$. The upper tree (in blue), represents the ground truth topology, while the lower tree (in red) represents the result from a pure SMC approach to building the topology with the given σ.

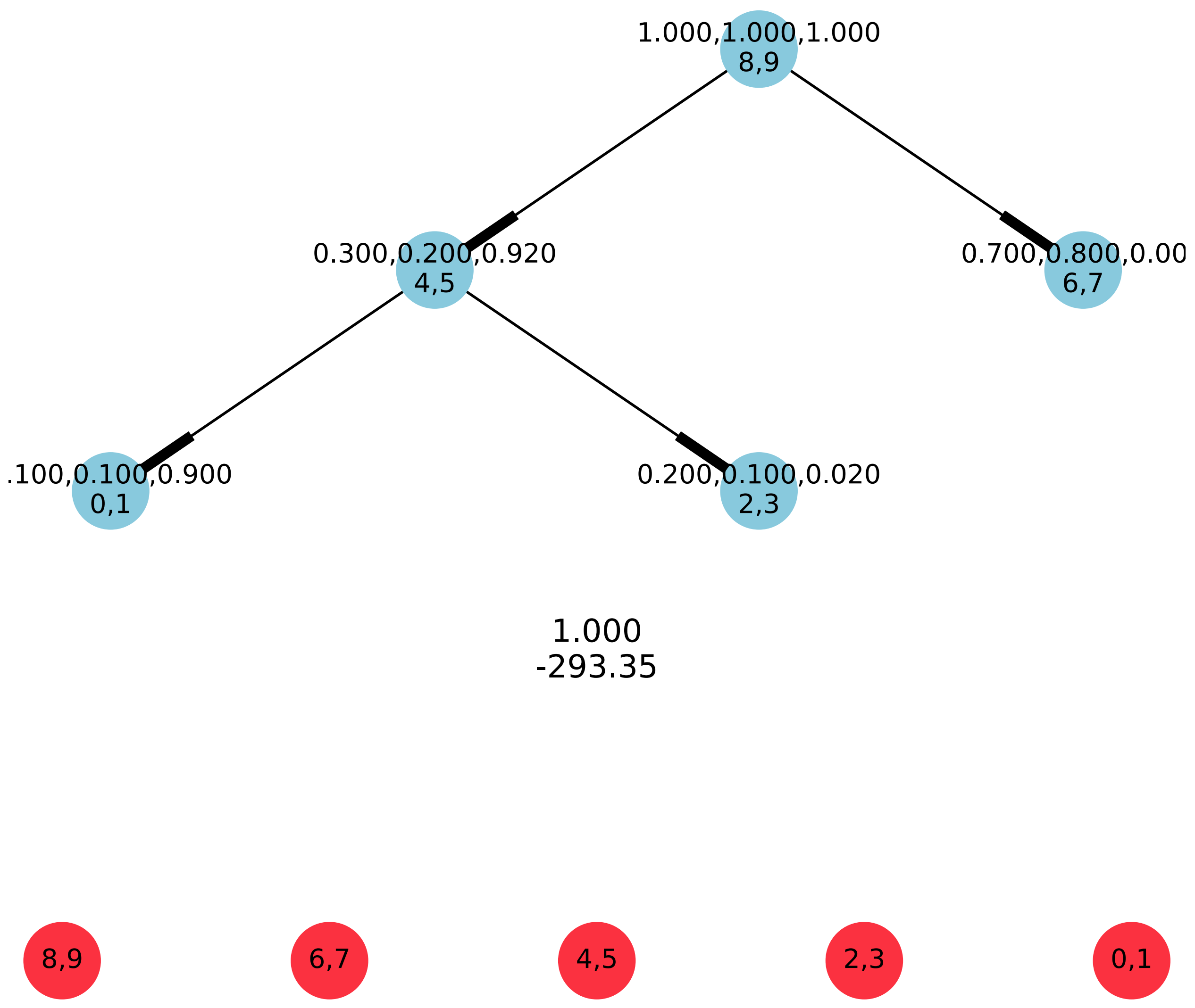

Figure S7: **SMC given a bad data ordering**. Graphical representation of how the data point ordering given to an SMC method can impact its end result when building up from the root. Data ordering given as $\sigma = (9, 8, \ldots, 1, 0)$. The upper tree (in blue), represents the ground truth topology, while the lower tree (in red) represents the result from a pure SMC approach to building the topology with the given σ.

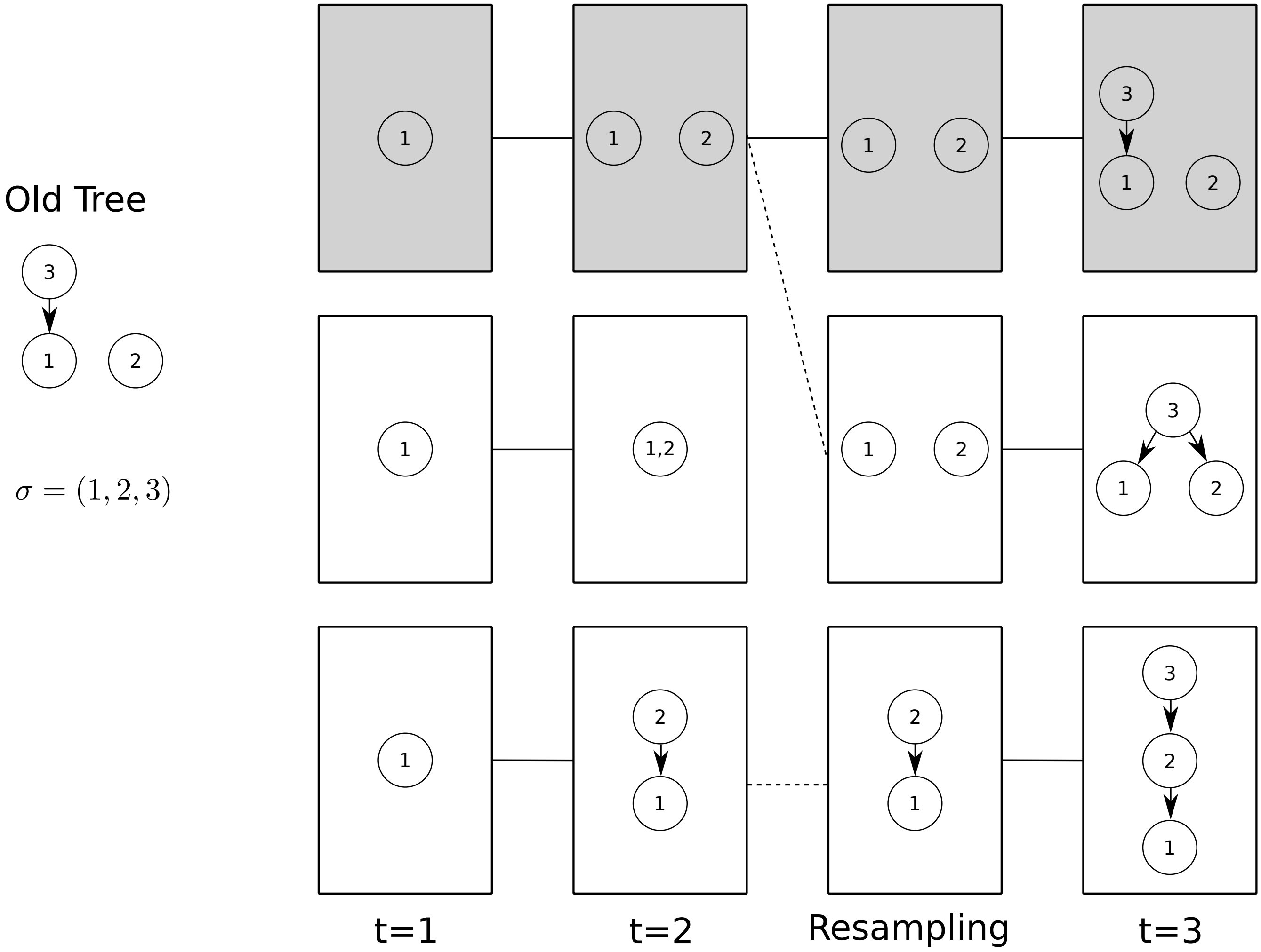

Figure S8: **Particle Gibbs SMC Sampler**. Diagram for the high-level overview of the PG-SMC sampling procedure. Conditional path which ensures the previous tree can be sampled at the final iteration shown in gray.

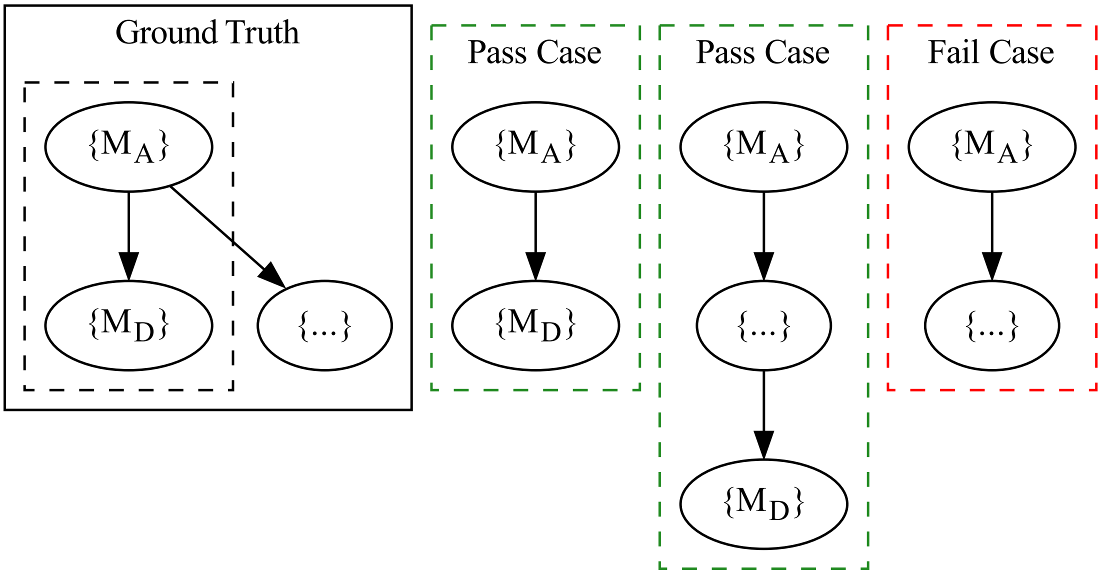

Figure S9: **Ancestor-Descendant reconstruction validity**. Graphical representation of how the ancestor-descendant relation metric defines a valid reconstruction. Curly brackets "{}" in nodes represent the set of SNVs assigned to that node. Ground truth box represents the true ancestor-descendant relationship between a single pair of SNVs $\{M_A, M_D\}$, where $M_A$represents an SNV that has an ancestor relationship to M_D.

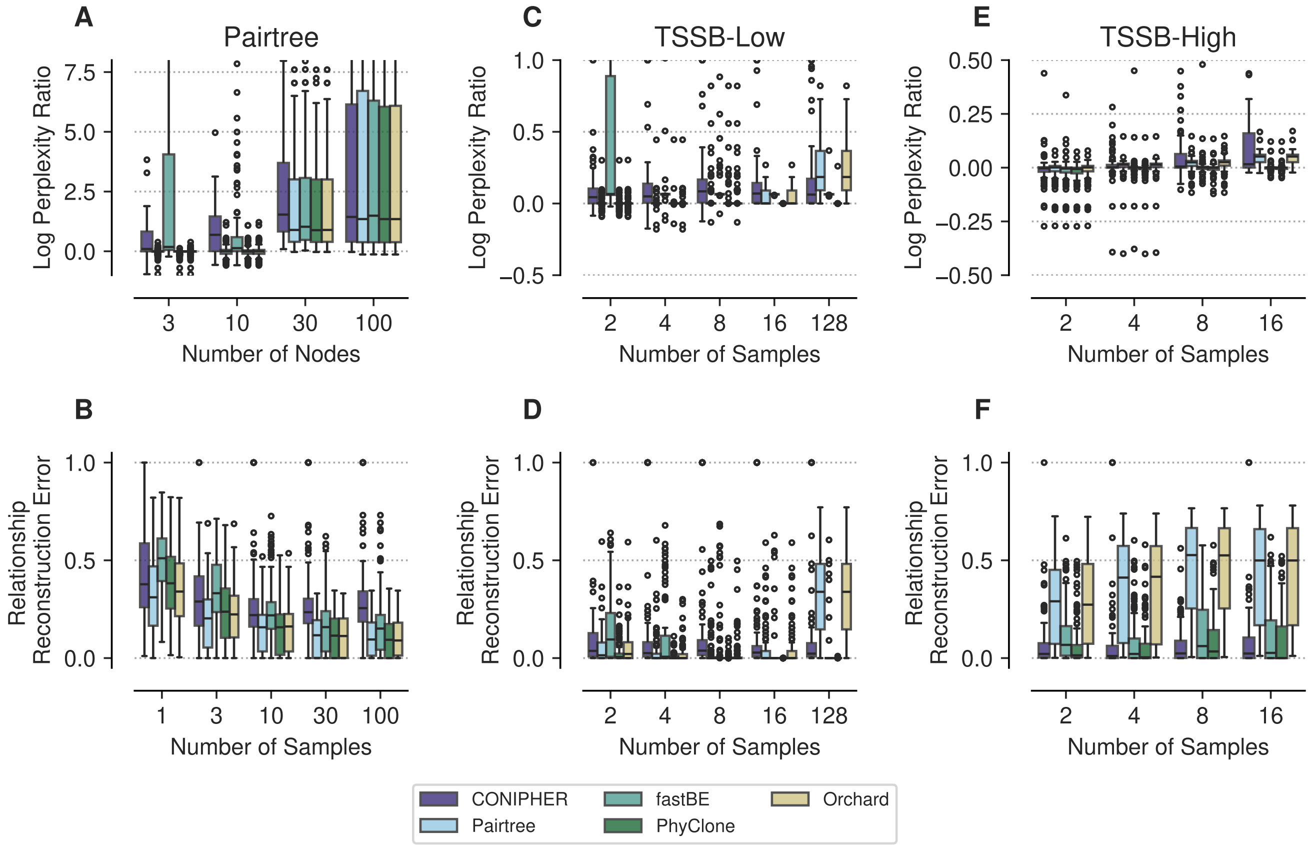

Figure S10: **Performance on noise-free synthetic data as measured by posterior metrics**. Method performance as measured by Log Perplexity Ratio and Relationship Reconstruction Error for: **A-B** Pre-clustered Pairtree, **C-D** Low SNV count TSSB (TSSB-Low), and **E-F** High SNV count TSSB (TSSB-High) datasets.

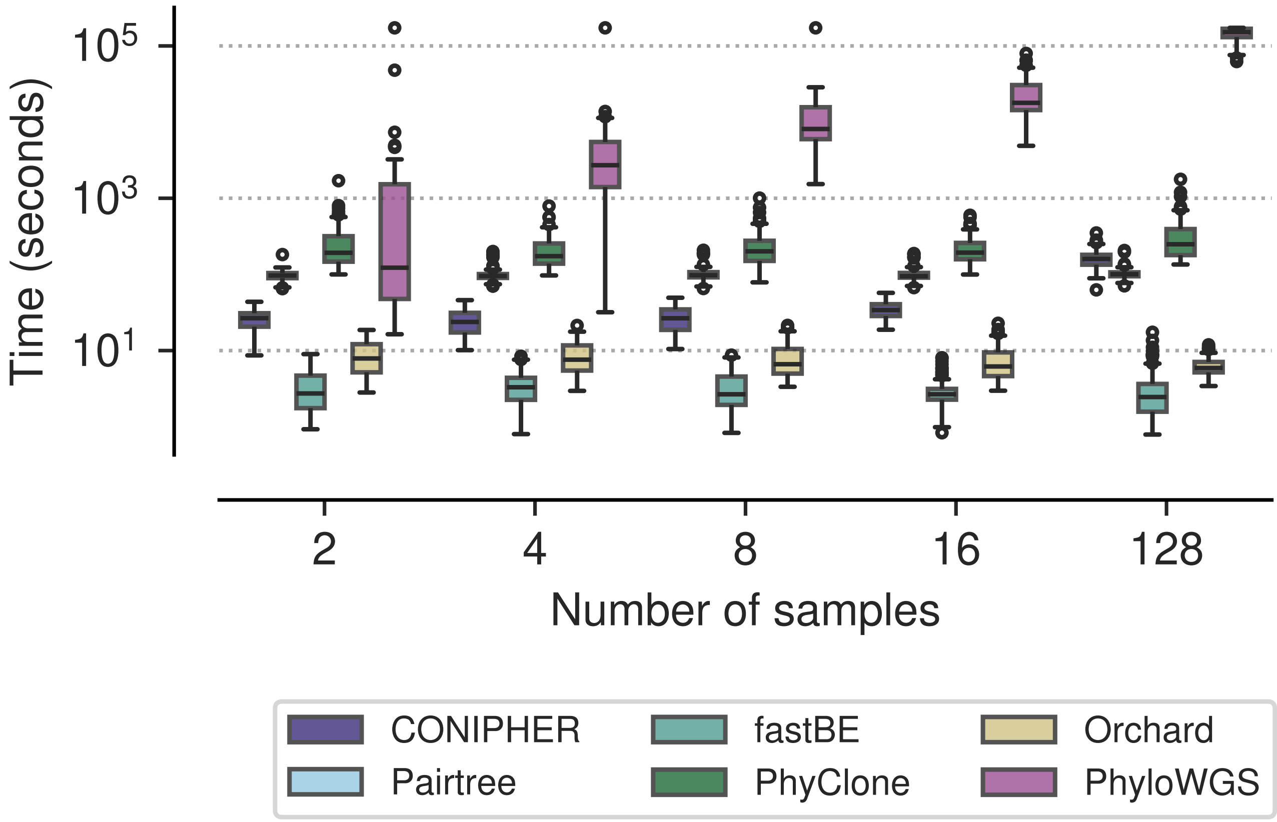

Figure S11: **Running time on small TSSB dataset by number of samples**. Running time in seconds for each method to analyse trials from the small TSSB dataset.

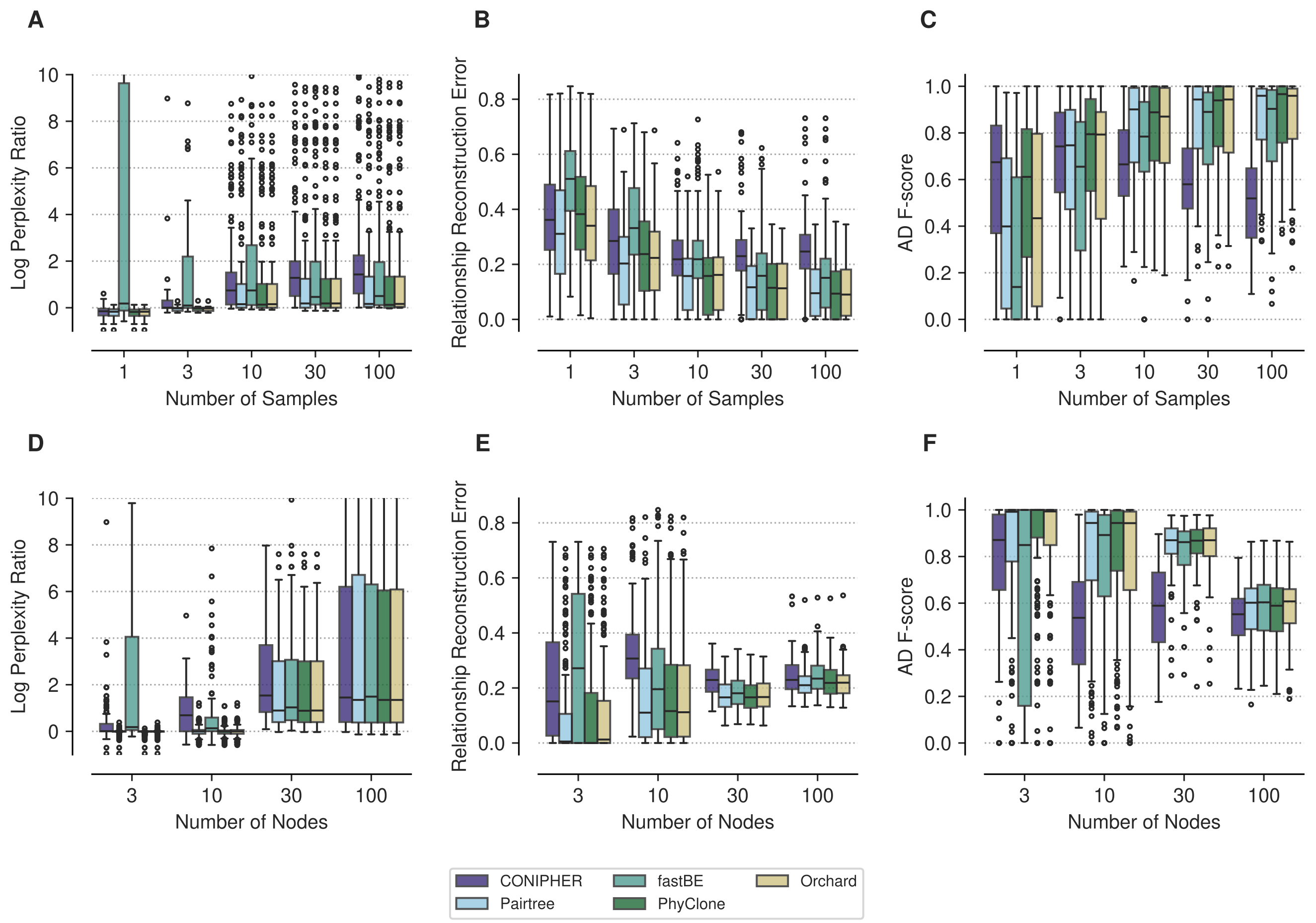

Figure S12: **Pre-clustered Pairtree data reconstruction performance, split by either increasing samples or nodes**. Method performance as measured by Log Perplexity Ratio, Relationship Reconstruction Error, and Ancestor-Descendant F-Score. Measures have been split to contrast performance across either number of samples or number of clonal nodes in the ground-truth tree.

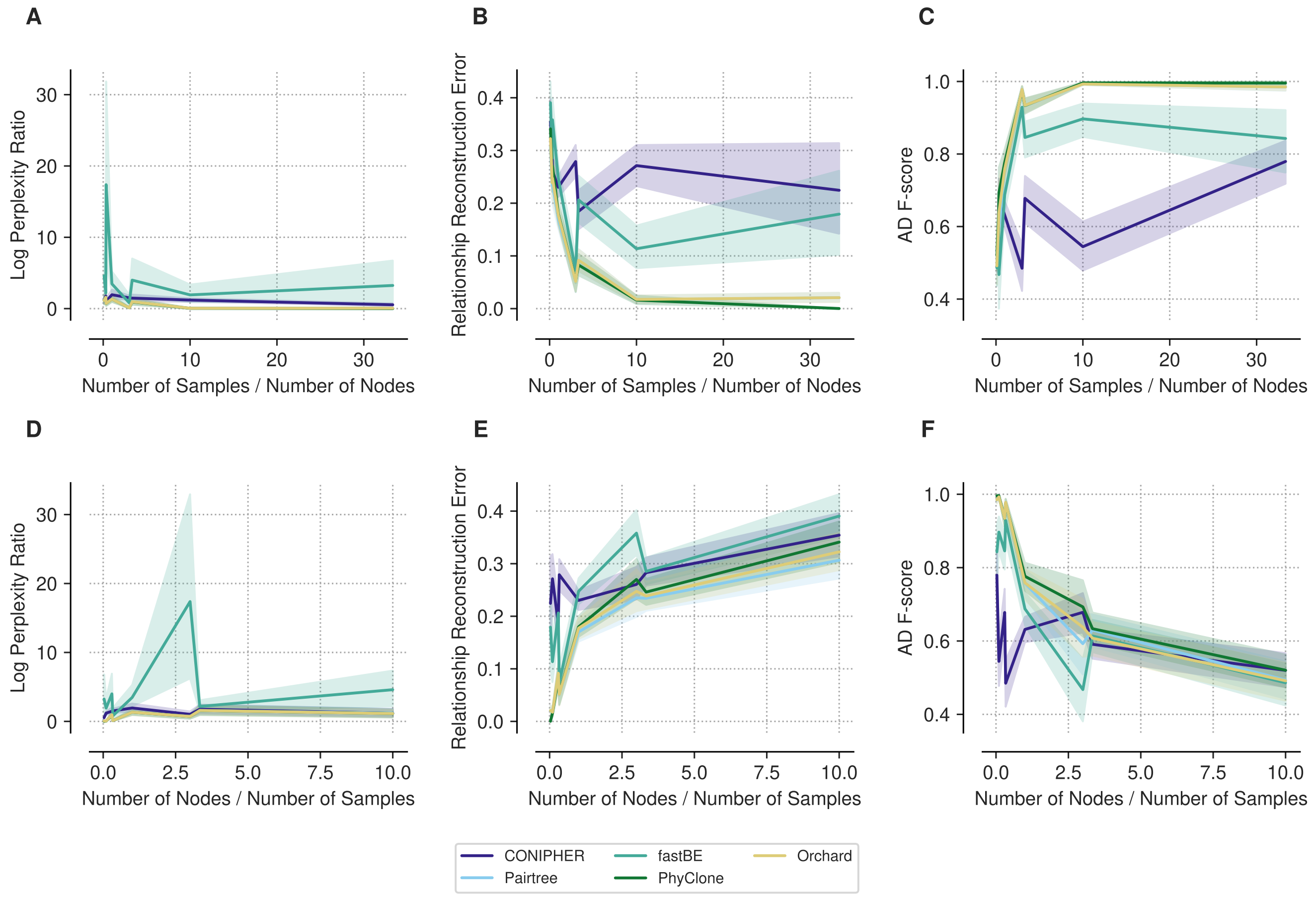

Figure S13: **Pre-clustered Pairtree data reconstruction performance as a function of sample vs. node ratios**. Method performance as measured by Log Perplexity Ratio, Relationship Reconstruction Error, and Ancestor-Descendant F-Score. Performance measures have been plotted as a function of sample versus node ratios, where nodes refer to the number of clonal nodes in the ground-truth tree.

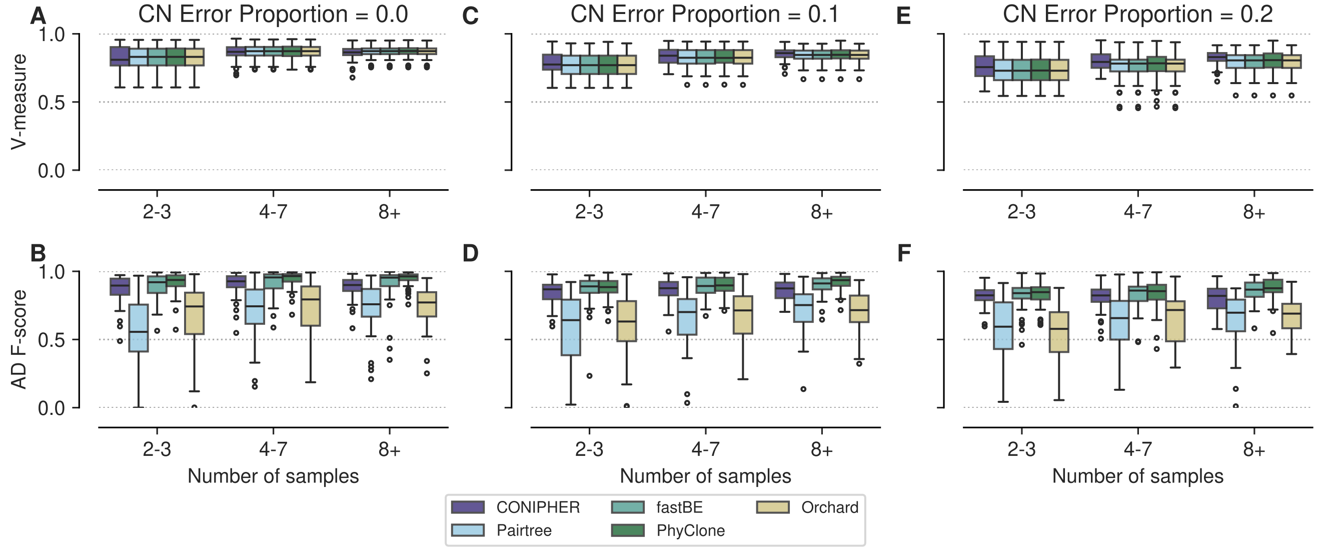

Figure S14: **Copy number perturbed data effect on method performance**. Method performance as measured by V-measure and Ancestor-Descendant F-Score on copy number perturbed data. Dataset selected is a perturbed version of the CONIPHER no-noise data, wherein a proportion, $p = \{0.0, 0.1, 0.2\}$, of the regions in each individual sample had the ground-truth copy number reading perturbed in one of the following ways: major or minor increases by one, major or minor decreases by one, both major and minor increase by one, or both major and minor decrease by one.

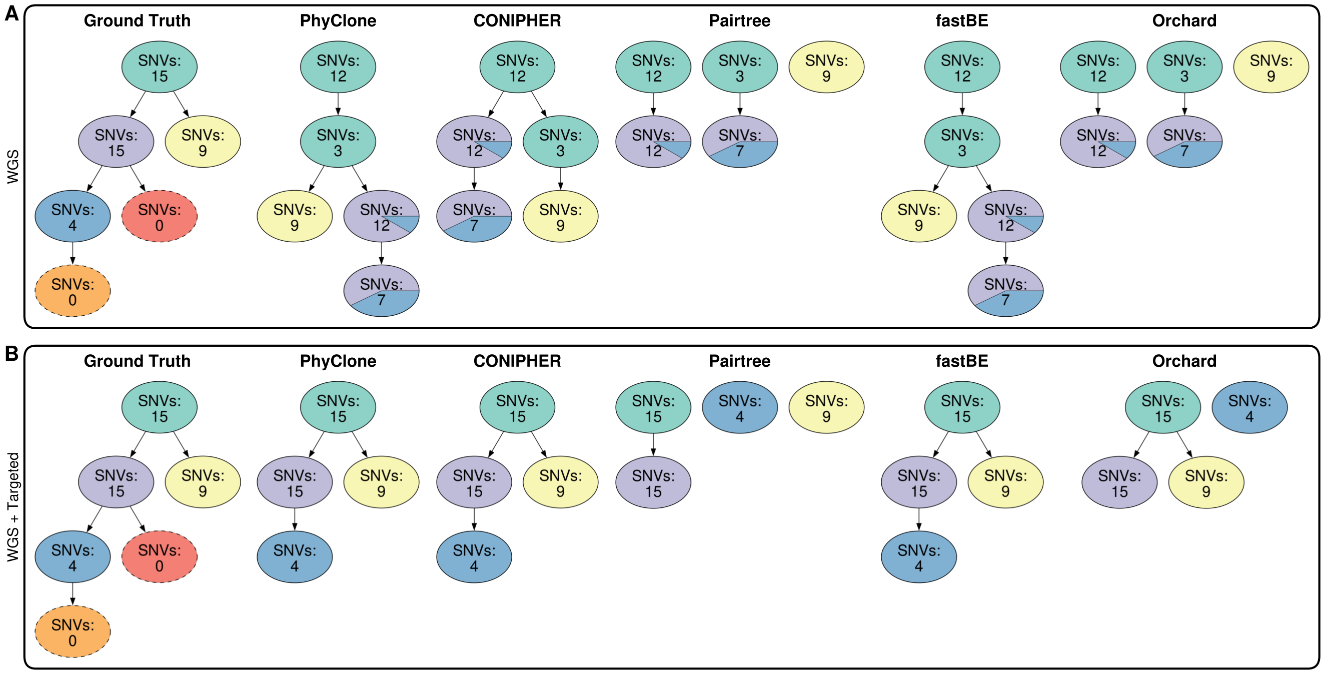

Figure S15: **HGSOC patient 2 clonal phylogenetic trees**. **A)** Predicted trees built using purely Whole Genome Sequencing (WGS) data. **B)** Predicted trees built using a combination of WGS and targeted deep sequencing data [2]. From left to right: Ground truth phylogenetic tree inferred from single cell and targeted deep sequencing data [2], nodes with a dashed border denote clones that are defined only by the absence of SNVs from the parent. Predicted trees built using: PhyClone with outlier modelling; CONIPHER; Pairtree; fastBE; and Orchard. Colours of nodes in method inferred trees correspond to the single nucleotide variant clonal (SNV) assignment from ground truth.

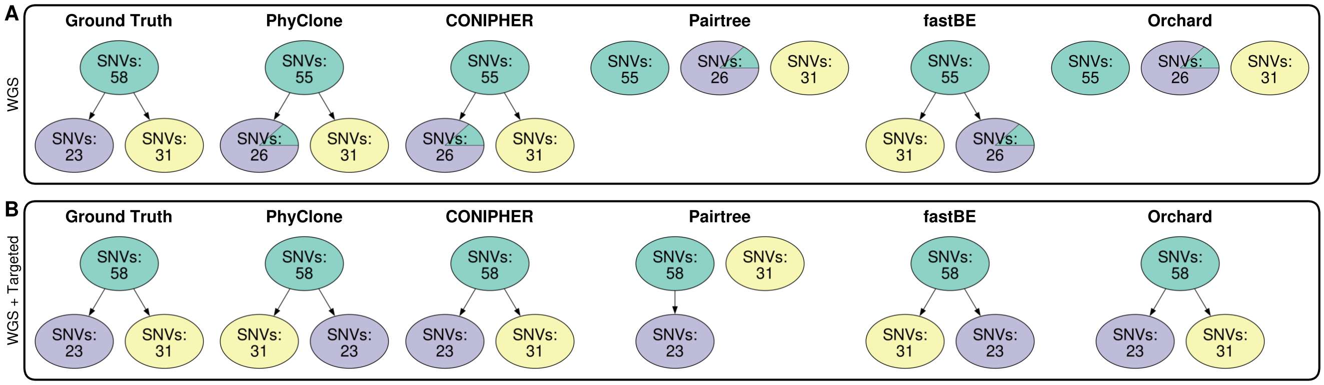

Figure S16: **HGSOC patient 9 clonal phylogenetic trees**. **A)** Predicted trees built using purely Whole Genome Sequencing (WGS) data. **B)** Predicted trees built using a combination of WGS and targeted deep sequencing data [2]. From left to right: Ground truth phylogenetic tree inferred from single cell and targeted deep sequencing data [2]. Predicted trees built using: PhyClone with outlier modelling; CONIPHER; Pairtree; fastBE; and Orchard. Colours of nodes in method inferred trees correspond to the single nucleotide variant clonal (SNV) assignment from ground truth.

S4 Supplementary Tables

Table S1: **High grade serous ovarian cancer data performance metrics**. Reported method performance metrics, V-measure (clustering accuracy) and Ancestor-Descendant F-Score (AD F-Score; phylogenetic reconstruction accuracy), were computed from two high grade serous ovarian cancer datasets [2] per patient: purely Whole Genome Sequencing (WGS) data, and a combination of WGS and targeted deep sequencing (WGS+T) data. Previously published ground truth phylogenetic trees were inferred from single cell and targeted deep sequencing data [2].

		V-Measure		AD F-Score	
	Dataset	WGS	WGS+T	WGS	WGS+T
Patient	Program				
2	CONIPHER	0.78	1.00	0.82	1.00
	Orchard	0.78	1.00	0.51	0.86
	Pairtree	0.78	1.00	0.51	0.64
	PhyClone	0.78	1.00	0.89	1.00
	fastBE	0.78	1.00	0.89	1.00
3	CONIPHER	0.73	0.86	0.70	0.80
	Orchard	0.73	0.86	0.34	0.32
	Pairtree	0.73	0.86	0.20	0.53
	PhyClone	0.85	1.00	0.82	0.93
	fastBE	0.73	0.86	0.73	0.80
9	CONIPHER	0.91	1.00	0.95	1.00
	Orchard	0.91	1.00	0.00	1.00
	Pairtree	0.91	1.00	0.00	0.60
	PhyClone	0.91	1.00	0.95	1.00
	fastBE	0.91	1.00	0.95	1.00

Supplementary Tables S2–S14: Benchmarking Performance Result Metrics
The following tables can be found in supplementary file: `Experiment_Performance_Metrics.xlsx`

- **S2**: TSSB-Low
- **S3**: TSSB-High
- **S4**: FS-CRP Loss
- **S5**: Pairtree
- **S6**: CONIPHER No-Noise
- **S7**: CONIPHER Noise
- **S8**: HGSOC WGS, patient 2
- **S9**: HGSOC WGS + Targeted, patient 2
- **S10**: HGSOC WGS, patient 3
- **S11**: HGSOC WGS + Targeted, patient 3
- **S12**: HGSOC WGS, patient 9
- **S13**: HGSOC WGS + Targeted, patient 9
- **S14**: Copy Number Error

Supplementary Tables S15–S27: Benchmarking Friedman-Nemenyi Analysis Results
The following tables can be found in supplementary file: `Experiment_Friedman_Nemenyi_Tests.xlsx`

- **S15**: TSSB-Low, Friedman
- **S16**: TSSB-Low, Nemenyi
- **S17**: TSSB-High, Friedman
- **S18**: TSSB-High, Nemenyi
- **S19**: FS-CRP Loss, Nemenyi
- **S20**: Pairtree, Friedman
- **S21**: Pairtree, Nemenyi
- **S22**: CONIPHER No-Noise, Friedman
- **S23**: CONIPHER No-Noise, Nemenyi
- **S24**: CONIPHER Noise, Friedman
- **S25**: CONIPHER Noise, Nemenyi
- **S26**: Copy Number Error, Friedman
- **S27**: Copy Number Error, Nemenyi

Supplementary Tables S28–S36: Benchmarking Friedman-Nemenyi Analysis Results
The following tables can be found in supplementary file: `Experiment_Posterior_Metrics.xlsx`

- **S28**: Pairtree Posterior Metrics
- **S29**: Pairtree Posterior Metrics, Friedman
- **S30**: Pairtree Posterior Metrics, Nemenyi
- **S31**: TSSB-Low Posterior Metrics
- **S32**: TSSB-Low Posterior Metrics, Friedman
- **S33**: TSSB-Low Posterior Metrics, Nemenyi
- **S34**: TSSB-High Posterior Metrics
- **S35**: TSSB-High Posterior Metrics, Friedman
- **S36**: TSSB-High Posterior Metrics, Nemenyi

References

[1] Kristiana Grigoriadis, Ariana Huebner, Abigail Bunkum, Emma Colliver, Alexander M. Frankell, Mark S. Hill, Nicolai J. Birkbak, Charles Swanton, Simone Zaccaria, and Nicholas McGranahan. Conipher: A computational framework for scalable phylogenetic reconstruction with error correction. *Protocol Exchange*, feb 2023.

[2] Andrew McPherson, Andrew Roth, Emma Laks, Tehmina Masud, Ali Bashashati, Allen W Zhang, Gavin Ha, Justina Biele, Damian Yap, Adrian Wan, Leah M Prentice, Jaswinder Khattra, Maia A Smith, Cydney B Nielsen, Sarah C Mullaly, Steve Kalloger, Anthony Karnezis, Karey Shumansky, Celia Siu, Jamie Rosner, Hector Li Chan, Julie Ho, Nataliya Melnyk, Janine Senz, Winnie Yang, Richard Moore, Andrew J Mungall, Marco A Marra, Alexandre Bouchard-Côté, C Blake Gilks, David G Huntsman, Jessica N McAlpine, Samuel Aparicio, and Sohrab P Shah. Divergent modes of clonal spread and intraperitoneal mixing in high-grade serous ovarian cancer. *Nature Genetics*, 48(7):758–767, may 2016.

[3] Andrew Roth, Jaswinder Khattra, Damian Yap, Adrian Wan, Emma Laks, Justina Biele, Gavin Ha, Samuel Aparicio, Alexandre Bouchard-Côté, and Sohrab P Shah. PyClone: statistical inference of clonal population structure in cancer. *Nature Methods*, 11(4):396–398, mar 2014.
